# Stakeholder Perspectives on the Integration of AI in Diabetes Care: Systematic Review of Qualitative Studies

**DOI:** 10.2196/105329

**Published:** 2026-09-22

**Authors:** Héctor Martínez-Martínez, Julia Martínez-Alfonso, Belén Sánchez-Rojo-Huertas, María Eugenia Visier-Alfonso, Fernando Sebastián-Valles, Ana Díez-Fernández, Ana Pérez-Moreno, Vicente Martínez-Vizcaíno

**Affiliations:** 1 Health and Social Research Center, Universidad de Castilla-La Mancha, Cuenca, Castilla-La Mancha, Spain; 2 Primary Care Center Zona 7-Feria, Servicio de Salud de Castilla La Mancha, Albacete, Castilla-La Mancha, Spain; 3 Hospital Universitario de Cuenca, Institute of Health Research of Castilla-La Mancha (IDISCAM), Cuenca, Castilla-La Mancha, Spain; 4 Daroca Primary Care Center, Madrid Health Service, Av. de Daroca, 4, Cdad. Lineal, Madrid, 28017, Spain, 34 600 20 92 09; 5 Instituto de investigación del Hospital Universitario de La Princesa. Universidad Autónoma de Madrid, Hospital Universitario de La Princesa, Madrid, Spain; 6 Faculty of Health Sciences, Universidad Autónoma de Chile, Talca, Araucanía, Chile

**Keywords:** artificial intelligence, diabetes mellitus, qualitative research, stakeholder perspectives, trust, digital health

## Abstract

**Background:**

AI is increasingly being integrated into diabetes care, with growing evidence supporting its potential to improve clinical decision-making, risk prediction, and self-management. However, the lived experiences, expectations, and concerns of those involved in its implementation have not been adequately synthesized.

**Objective:**

This study aims to synthesize qualitative evidence on the perspectives of patients, caregivers, health care professionals (HCPs), and other stakeholders regarding the integration of AI in diabetes management.

**Methods:**

We searched MEDLINE via PubMed, Web of Science, Scopus, CINAHL, and PsycINFO from inception to February 17, 2026. Eligible studies examined the perspectives of adult patients, caregivers, health care professionals, or administrators on AI-enabled tools for diabetes management, self-management, clinical decision support, or the prevention of complications, and used qualitative methods or reported a separately analyzable qualitative component. Tools required an identifiable data-driven function for prediction, classification, recommendation, personalization, or decision support. Studies focused exclusively on image-based diagnosis, technical validation, or digital tools without an identifiable AI component were excluded. Two reviewers (HM-M and JM-A) independently screened studies and extracted data. Methodological limitations were assessed using the Joanna Briggs Institute (JBI) checklist and the Cochrane Qualitative Methodological Limitations Tool (CAMELOT). Findings were synthesized using thematic synthesis. Confidence was assessed using GRADE-CERQual. A sensitivity analysis excluded questionnaire-based qualitative evidence.

**Results:**

Fourteen studies published between 2023 and 2025 were included, representing at least 738 participants across 9 countries. Participants included individuals with type 1 or type 2 diabetes, family caregivers, doctors, nurses, specialists, administrators, and other health care staff. Studies evaluated large language models, AI-enabled mobile applications and wearables, glucose-prediction systems, and clinical decision-support tools. Exposure ranged from direct use of functioning systems to evaluation of prototypes, wireframes, and hypothetical applications. Four analytical themes and 13 subthemes were identified. Stakeholders perceived that AI could support preventive and individualized care, education, self-management, and decision-making. Concerns included accuracy, bias, privacy, accountability, increased workload, caregiver burden, loss of professional autonomy, and erosion of human-centered care. Participants emphasized explainability, intuitive design, integration with existing systems, tailored training, and continued access to human support. Eleven findings were rated as high confidence and 2 as moderate confidence.

**Conclusions:**

Although stakeholders perceived AI to be useful for diabetes care, these qualitative findings do not demonstrate clinical effectiveness, safety, or improved patient outcomes. Evidence was limited by heterogeneity in AI modalities, stakeholder groups, diabetes contexts, and technology exposure, as well as demographic imbalance, restricted reporting of researcher reflexivity, and reliance on prototype or hypothetical systems. Implementation should prioritize transparent design, clinical validation, data governance, human oversight, and tailored support, while preserving professional judgment and person-centered relationships.

## Introduction

### Background

AI is rapidly emerging as a transformative force in modern medicine and is promising for reshaping clinical workflows, supporting personalized care, and improving patient outcomes [1,2]. Although its application has historically focused on image-centric fields such as radiology and dermatology [3,4], AI is increasingly permeating the management of chronic conditions that require continuous data analysis and complex decision-making processes [5]. Among these, diabetes mellitus (DM) is particularly well suited to AI-supported care because its daily management requires the continuous interpretation of large volumes of data, including glycemic patterns, insulin dosing, dietary intake, physical activity, and other behavioral and physiological variables [6-8].

In recent years, diabetes care has evolved from reactive monitoring to proactive and increasingly automated management approaches [9]. AI-driven technologies, particularly machine learning (ML) algorithms, now support advanced tools such as hybrid closed-loop systems, which automate insulin delivery in response to real-time glucose sensor data [10,11]. AI models are also being applied beyond therapeutic delivery to risk prediction. This offers the potential to predict adverse events such as hypoglycemia or diabetic ketoacidosis before they occur and to identify individuals at high risk of disease progression [12]. Collectively, these advancements may substantially reduce the cognitive and emotional burden of diabetes self-management, which is often described as a full-time job for patients and their caregivers [13].

Despite the enthusiasm, the transition from theoretical models to clinical practice has revealed important limitations, as algorithmic precision does not necessarily ensure clinical effectiveness or safety. Evidence indicates that automated systems are susceptible to routine human errors and raise complex ethical concerns regarding data privacy and the medicalization of domestic life [14,15]. Furthermore, the successful implementation of these innovations requires a clear understanding of 3 key dimensions: their benefits for individuals, the barriers that limit their adoption, and their potential future applications [16]. Currently, many ML models have not demonstrated clear clinical superiority over traditional statistical methods and remain constrained by a lack of external validation and poor generalizability [6].

The successful adoption of AI in diabetes management depends not only on algorithmic performance but also on the trust and acceptance of the stakeholders involved. Although numerous quantitative studies have evaluated the clinical efficacy of these tools [17,18], evidence regarding human experience, specifically the perceptions, barriers, and facilitators of their adoption, remains fragmented and often limited to specific devices or user groups [19].

### Objectives

The primary objective of this systematic review was to synthesize qualitative evidence to provide an in-depth understanding of the perspectives of patients, caregivers, and health care professionals (HCPs) regarding the use of AI in diabetes management, glycemic control, and risk prevention.

## Methods

### Overview

This systematic review was performed in accordance with the methodological framework established by the Cochrane Qualitative and Implementation Methods Group (CAMELOT) [20], and the protocol was prospectively registered in the International Prospective Register of Systematic Reviews (PROSPERO) database (registration number: CRD420251010095). The reporting of this review complies with the Enhancing Transparency in Reporting the Synthesis of Qualitative Research (ENTREQ) statement (Multimedia Appendix 1) [21].

### Search Strategy and Selection Criteria

A systematic literature search was conducted by 2 reviewers (HM-M and JM-A) across 5 major databases: MEDLINE (via PubMed), Web of Science, Scopus, CINAHL, and PsycInfo, covering the period from their inception to February 17, 2026. To develop the search strategy, we used the Sample, Phenomenon of Interest, Design, Evaluation, and Research type (SPIDER) framework (Multimedia Appendix 2) [22] to identify relevant qualitative and mixed methods research.

The search terms combined concepts related to diabetes management, AI, and user perceptions. The specific search syntax was tailored to the requirements of each database (Multimedia Appendix 3). Eligible studies were required to meet the following criteria: (i) provide a qualitative analysis of perceptions, barriers, or facilitators related to the use of AI tools for diabetes management or risk of complications; (ii) include adult patients, caregivers, or HCPs as the study population; and (iii) use qualitative data collection methods, such as semistructured interviews or focus groups, requiring a formal qualitative data analysis approach. For mixed methods studies, inclusion depended on the availability of separate, distinct qualitative findings. Only articles published in English or Spanish were considered.

For this review, an AI-enabled tool was operationally defined as a digital system that the primary study explicitly described as using AI, ML, deep learning, natural language processing, generative AI, or an adaptive data-driven algorithm to perform prediction, classification, recommendation, personalization, or decision support beyond fixed-rule data capture, storage, display, or communication. Apps and wearable devices were eligible only when an identifiable AI-enabled function was integral to the phenomenon explored; general mobile health (mHealth), telehealth, or monitoring tools without an AI component were excluded. Studies that focused exclusively on the diagnosis of complications through images, such as diabetic retinopathy, were excluded because this review focused on AI embedded in ongoing diabetes management, self-management, and clinical decision support. Image interpretation represents a distinct diagnostic pathway with different users and workflows that warrants separate synthesis. However, studies that included image analysis as one component of a broader diabetes-management tool remained eligible. Studies focusing solely on technical validation, as well as gray literature, conference abstracts, protocols, dissertations, and theses, were excluded.

### Research Screening and Data Extraction

Following the search, the citations were imported into Rayyan software (Rayyan Systems Inc) [23], and duplicates were removed. The screening process was conducted in 2 stages by 2 independent reviewers (HM-M and JM-A). First, titles and abstracts were assessed against the inclusion criteria. The full texts of potentially relevant records were subsequently assessed to determine final eligibility. Any discrepancies arising during this process were resolved through discussion or, if necessary, by consulting a third reviewer (VM-V).

### Quality Assessment

The methodological rigor and risk of bias of the included studies were evaluated independently by 2 reviewers (HM-M and JM-A), with disagreements resolved through consensus. To ensure a comprehensive assessment, 2 complementary tools were used. First, the Joanna Briggs Institute (JBI) critical appraisal checklist for qualitative research (Table 1) was used to assess the trustworthiness of the studies [24]. Additionally, the CAMELOT tool (Multimedia Appendix 4) [25] was applied to identify specific limitations, which informed the subsequent Grading of Recommendations Assessment, Development, and Evaluation–Confidence in the Evidence From Reviews of Qualitative Research (GRADE-CERQual) assessment of confidence in the review findings [26].

**Table 1.** Joanna Briggs Institute (JBI) critical appraisal checklist for qualitative research.

Author and year	Q1^a^	Q2^b^	Q3^c^	Q4^d^	Q5^e^	Q6^f^	Q7^g^	Q8^h^	Q9^i^	Q10^j^
Yoon, Sungwon et al [27], 2025	Y^k^	Y	Y	Y	Y	U^l^	U	Y	Y	Y
Liaw, Winston R. et al [28], 2023	Y	Y	Y	Y	Y	U	U	Y	Y	Y
Jairoun, Ammar Abdulrahman et al [29], 2024	Y	Y	Y	Y	Y	U	U	Y	Y	Y
Alanzi, Turki M. et al [30], 2025	Y	Y	Y	Y	Y	U	U	Y	Y	Y
Yoon, Sungwon et al [31], 2024	Y	Y	Y	Y	Y	U	U	Y	Y	Y
Robinson, Renee et al [32], 2023	Y	Y	Y	Y	Y	U	U	Y	Y	Y
Lim, Phei-Ching et al [33], 2025	Y	Y	Y	Y	Y	U	U	Y	Y	Y
Yoon, Sungwon et al [34], 2024	Y	Y	Y	Y	Y	U	U	Y	Y	Y
Krisnan, Logeswary et al [35], 2025	Y	Y	Y	Y	Y	U	U	Y	Y	Y
Samimi, Reza et al [36], 2025	Y	Y	Y	Y	Y	U	U	Y	Y	Y
Klemme, Isabel et al [37], 2023	Y	Y	Y	Y	Y	U	U	Y	Y	Y
Stawarz, Katarzyna et al [38], 2023	Y	Y	Y	Y	Y	U	U	Y	Y	Y
Alzghaibi, Haitham [39], 2025	Y	Y	Y	Y	Y	U	U	Y	Y	Y
Roy, Mrinmoy et al [40], 2025	Y	Y	Y	Y	Y	U	U	Y	Y	Y

a Q1: Is there congruity between the stated philosophical perspective and the research methodology?

b Q2: Is there congruity between the research methodology and the research question or objectives?

c Q3: Is there congruity between the research methodology and the methods used to collect data?

d Q4: Is there congruity between the research methodology and the representation and analysis of data?

e Q5: Is there congruity between the research methodology and the interpretation of results?

f Q6: Is there a statement locating the researcher culturally or theoretically?

g Q7: Is the influence of the researcher on the research, and vice-versa, addressed?

h Q8: Are participants, and their voices, adequately represented?

i Q9: Is the research ethical according to current criteria or, for recent studies, and is there evidence of ethical approval by an appropriate body?

j Q10: Do the conclusions drawn in the research report flow from the analysis, or interpretation, of the data?

k Y: yes.

l U: unclear

### Data Extraction and Synthesis

Two reviewers (HM-M and BS-R-H) independently extracted data using a standardized form. Extracted variables included study characteristics, participant characteristics, diabetes type, stakeholder role, data collection and analysis methods, AI function, level of participant exposure, and the analytical themes to which each study contributed (Table 2 and Multimedia Appendix 5). Raw participant numbers were not used to weight study contributions. Instead, contribution to the synthesis was assessed according to relevance, contextual richness, and depth of qualitative data. Open-ended questionnaire responses were therefore treated as less contextually rich than interview, focus-group, or longitudinal co-design data when assessing adequacy.

**Table 2.** Description of the included studies

Author, year and country	Characteristics of the participants	Data collection	Method of analysis (framework, software)	AI approach	Place and recruitment method
Yoon, Sungwon et al [27], 2025, Singapore	n=50 Patients (T2DM):^a^ n=25 Family members: n=25 Gender (patients): men: 15 (60%); women: 10 (40%) Age (patients): mean 53 (range 31‐68; years)	Semistructured interviews via online video conferencing. Mock wireframes were used to facilitate feedback on app features.	Inductive thematic analysis (constant-comparative method; NVivo 12 software).	Aim: to explore acceptability and feature preferences for family-based AI support. AI object: specific tool (EMPOWER app - FAMILY module). Features: personalized behavioral nudges and AI-driven food recognition.	Community health center (Singapore) and follow-up (callback) of participants from previous mHealth trials. Purposive sampling.
Liaw, Winston R. et al [28], 2023, United States	n=22 Clinicians (n=16): physicians (n=14), ^b^NP or PA^c^ (n=2); HCPs (n=6): nurse (n=1), behavioral spec. (n=1), social worker (n=1), admin or other ( n=3). Gender: women: 13 (59.09%); men: 8 (36.36%); prefer not to answer: 1 (4.54%) Age (years): not reported.	Semistructured interviews conducted via Zoom or telephone.	Thematic analysis (inductive-deductive approach; NVivo software).	Aim: to assess clinician and staff usage and perceptions of diabetes prediction tool. AI object: AI-based risk stratification model integrated into the EHR^d^ to predict uncontrolled diabetes.	Three federally qualified health centers in Houston, Texas. Purposive sampling.
Jairoun, Ammar Abdulrahman et al [29], 2024, United Arab Emirates	n=25 Endocrinologists (n=16), diabetologists (n=9). Gender: men: 15 (60%); women: 10 (40%). Age (years): mean 38 (SD 2).	Semistructured in-depth interviews.	Thematic analysis (Braun & Clarke framework; not reported).	Aim: to investigate the perceived benefits and risks of ChatGPT in diabetes and metabolic disease management. AI object: ChatGPT (Generative AI).	Private and public hospitals (primary and secondary care) in the UAE. Purposive sampling.
Alanzi, Turki M. et al [30], 2025, Saudi Arabia	n=25 Patients with DM^e^ (type not disaggregated) Gender: men: 14 (56%); women: 11 (44%). Age (years): mean 32.4 (range 19‐50).	Experimental qualitative study. Participants used ChatGPT for 1 week, followed by semistructured interviews.	Thematic analysis (Braun & Clarke framework; NVivo software).	Aim: to analyze the impact of ChatGPT on DM self-management. AI object: ChatGPT (free version 3.5).	King Fahad University Hospital, Saudi Arabia. Purposive sampling.
Yoon, Sungwon et al [31], 2024, Singapore	n=33 Patients (T2DM). Gender: men: 23 (70%); women: 10 (30%). Age (years): mean 56 (range 42‐66).	Semistructured interviews conducted by videoconferencing. A mock-up app wireframe was used to facilitate feedback.	Thematic analysis (inductive approach; NVivo 12 software).	Aim: to explore the acceptability of app-based MI and preferences for MI module features. AI object: EMPOWER mobile application (MI^f^ module).	Public primary care clinics (polyclinics) in Singapore. Purposive sampling.
Robinson, Renee et al [32], 2023, United States	n=47 Patients (n=41): T1DM:^g^ 17 (41.5%); T2DM: 24 (58.5%). HCPs: nurse case managers, pharmacists, physicians, and an endocrinologist (n=6). Gender (patients): women: 21 (51.2%); men: 19 (46.3%); prefer not to answer: 1 (2.4%). Age (patients): mean 48.4 (SD 20.4) years.	Mixed qualitative methods: focus groups and interviews.	Grounded theory and thematic content analysis (CFIR^h^; QDA Miner software).	Aim: to understand patient information needs and design comprehensible transparency for AI or ML^i^ in health care. AI object: AI or ML models for Glc^j^ prediction and insulin dosing (hypothetical transparency displays).	Alaska, Idaho, and Virginia (USA). Purposive sampling.
Lim, Phei-Ching et al [33], 2025, Malaysia	n=19 HCPs:^k^ pharmacists (n=8), endocrinologists (n=4), family medicine specialists (n=3), dietitians (n=2), diabetes educators (n=2). Gender: not reported. Age (years): range 30‐69.	Semistructured interviews conducted via video conferencing.	Thematic analysis (Braun & Clarke framework; NVivo 14 software).	Aim: to explore HCPs’ perspectives on AI-based mobile apps for diabetes education and behavioral management. AI object: AI-enabled mobile applications (General concept).	Health care settings across Malaysia. Purposive sampling.
Yoon, Sungwon et al [34], 2024, Singapore	n=13 Endocrinologists (n=13). Gender: men: 8 (62%); women: 5 (38%). Age (years): 31‐40 (n=6), 41‐50 (n=6), >50 (n=1).	Focus groups (n=3 sessions) organized by professional seniority.	Reflexive thematic analysis (Braun & Clarke framework; NVivo 12 software).	Aim: to assess the utility, impact, and adoption challenges of an AI-enabled prescription advisory tool for T2DM management. AI object: AI-enabled Prescription Advisory	Singapore General Hospital. Purposive sampling.
Krisnan, Logeswary et al [35], 2025, Malaysia	n=17 Patients (T2DM). Gender: women: n=14 (82.4%); men: n=3 (17.6%). Age (years): 26‐35: n=3 (17.6%); 36‐49: n=9 (52.9%); 50‐65: n=5 (29.4%).	Semistructured in-depth interviews.	Thematic content analysis (Braun & Clarke framework; ATLAS.ti software).	Aim: to explore patient perceptions and attitudes toward AI tools for diabetes care in public primary care settings. AI object: general AI applications in health care (remote monitoring, diagnosis, and patient alert systems).	Endocrine Clinic, Hospital Tengku Ampuan Rahimah, Malaysia. Purposive sampling.
Samimi, Reza et al [36], 2025, Belgium	n=30 HCPs: nurses (n=23; 76.7%), physicians (n=2; 6.7%), paramedics (n=2; 6.7%), others (n=3; 10%). Gender: women: n=25 (83.3%); men: n=5 (16.7%). Age (years): 20‐30 (n=4; 13.3%), 30‐40 (n=13; 43.3%), 40‐50 (n=8; 26.7%), 50‐60 (n=5; 16.7%).	Mixed methods study: user study with 4 interactive tasks and qualitative feedback via open-ended questions.	Thematic analysis (hybrid deductive-inductive approach; not reported).	Aim: to evaluate trust and actionability of evidence-based explanations for GLC-related risk prediction. AI object: visual-conversational DSS^l^ prototype (hybrid LLM^m^ architecture).	Online study (Prolific platform). Participants from the UK (n=21), USA (n=7), and Canada (n=2). Purposive sampling.
Klemme, Isabel et al [37], 2023, DEU^n^	n=24 Patients (T1DM). Gender: men: n=12 (50%); women: n=12 (50%). Age: range 23‐81 years.	Semistructured interviews (face-to-face and via video conferencing).	Inductive content analysis and deductive axial coding (RRI^o^ vision assessment framework; ATLAS.ti software).	Aim: to explore patient perspectives and visions regarding desired features and content of future diabetes applications. AI object: future features in mobile apps and smartwatches (eg, Glc prediction, AI-driven photo analysis for BU^p^ calculation).	Diabetologist practice in Germany. Purposive sampling.
Stawarz, Katarzyna et al [38], 2023, United Kingdom	n=15 Adults with T1DM. Gender: men: n=11 (73.3%); women: n=4 (26.7%). Age: mean 36.4 (SD 11.4); range 24‐69 years.	Longitudinal co-design (9 months): initial interviews followed by 6 workshops and prototype reviews.	Reflexive thematic analysis (sensemaking theory; NVivo software).	Aim: to identify opportunities for human-centered ML to support T1DM decision-making in unexpected situations. AI object: co-designed concepts for outlier or nonroutine situation support.	Bristol and Cardiff, United Kingdom. Purposive sampling.
Alzghaibi, Haitham [39], 2025, Saudi Arabia	n=418 Patients. T1DM: n=248 (59.3%); T2DM: n=170 (40.7%). Gender: men: n=265 (63.4%); women: n=153 (36.6%). Age (years): 18‐25: n=23 (5.5%); 26‐35: n=68 (16.3%); 36‐45: n=63 (15%); 46‐55: n=84 (20.1%); 56‐65: n=144 (34.4%); 66‐75: n=56 (13.4%)	Mixed methods study: structured digital questionnaire with Likert-scale items and open-ended responses.	Thematic analysis (qualitative responses; SPSS v29 and R v4.3.0).	Aim: to investigate perceptions of AI-integrated wearables for self-management. AI object: AI-integrated wearable devices (CGMs^q^, smart insulin pens, smartwatches, and mHealth apps).	Online platforms and patient groups in Saudi Arabia. Purposive sampling.
Roy, Mrinmoy et al [40], 2025, India	n=not specified. HCPs (administrators: n=not specified; patients: n=not specified).	Semistructured interviews using an adaptable guide with open-ended questions.	Thematic analysis (Braun & Clarke framework; R software).	Aim: to explore the adoption and impact of AI-based diagnostic interventions across the patient journey. AI object: AI-based diagnostic tools, CGM systems, smart wearables, and AI-powered chatbots.	15 multispecialty hospitals and clinics in Maharashtra and Karnataka, India. Purposive sampling based on existing AI adoption.

a T2DM: type 2 diabetes mellitus.

b NP: nurse practitioner.

c PA: physician assistant.

d EHR: electronic health record.

e DM: diabetes mellitus.

f MI: motivational interviewing

g T1DM: type 1 diabetes mellitus.

h CFIR: consolidated framework for implementation research.

i ML: machine learning.

j Glc: glucose.

k HCP: health care professional.

l DSS: decision support system.

m LLM: large language model.

n DEU: Germany.

o RRI: responsible research and innovation.

p BU: bread units

q CGM: continuous glucose monitoring.

We adopted a thematic synthesis approach [41] to integrate the qualitative findings. Two reviewers (HM-M and JM-A) performed line-by-line coding of the findings reported in the primary studies. These codes were initially organized into descriptive themes grounded in the data. These themes were subsequently interpreted to generate overarching analytical themes that provided new insights into the phenomenon under study. Disagreements were resolved through discussion and, when necessary, arbitration by a third reviewer (VM-V). To examine the influence of questionnaire-based qualitative evidence, we repeated the study-to-theme mapping after excluding the cross-sectional study in which qualitative data consisted of open-ended questionnaire responses [39].

### Confidence in the Evidence Obtained

Confidence in each synthesized finding was appraised using GRADE-CERQual (Table 3 and Multimedia Appendix 6) [26,42].

**Table 3.** GRADE-CERQual^a^ (confidence in the evidence from reviews of qualitative research). Summary of qualitative findings.

Summarized review finding	GRADE-CERQual assessment of confidence	Explanation of GRADE-CERQual assessment	References
Enhancing clinical utility: prevention, management, and performance			
Prevention, early diagnosis, and precision			
AI is viewed as a potential tool to support preventive care, identify subtle patterns, and provide an additional safety net for clinicians.	High confidence	Minor methodological concerns (universal unclear reflexivity) did not substantially alter the coherence of the finding, which is supported by 6 studies with sufficient qualitative richness.	[28,29,33,34,36,40]
Holistic and individualized management			
Stakeholders perceived that AI could support personalized care by incorporating patient-specific and nonclinical factors and enabling more frequent monitoring.	High confidence	There is a strong fit between the data and the finding across diverse clinical settings, offsetting minor reflexivity and transferability concerns.	[27-29,34,40]
Time management and clinical safety			
AI is anticipated to support consultation efficiency, reduce clinic congestion, and reinforce education outside traditional encounters.	High confidence	It is supported by rich data from 7 studies; minor methodological limitations (reflexivity and demographics) did not materially reduce coherence.	[28,29,31,33-36]
The role of technology in patient empowerment and self-management			
Personalized education and awareness			
AI is regarded as a potentially personalized educational resource that could filter information and support patient ownership.	High confidence	The educational roles described are coherent and directly relevant to self-management, mitigating minor methodological reporting concerns.	[27,28,30,31,33,40]
Decision-making support and real-life adaptation			
Stakeholders perceived AI as a potential safety net for out-of-routine situations such as celebrations, illness, or intense exercise.	High confidence	Longitudinal and conceptual data provide coherent insights into daily challenges. Minor methodological concerns were offset by data adequacy.	[27,30,33,38-40]
Motivation, discipline, and emotional well-being			
Some stakeholders perceived AI as a companion that might help address mental barriers and diabetes-related stress.	Moderate confidence	Confidence was downgraded to moderate due to moderate concerns in data adequacy (lower richness compared to technical subthemes) and minor concerns in coherence due to variations in how participants express their feelings.	[27,30,31,33]
Support network: family, social, and clinical connection			
AI-enabled shared records are expected to strengthen communication among patients, family caregivers, and clinical teams.	High confidence	The supporting studies provided sufficiently rich accounts of caregiver communication needs, offsetting minor methodological concerns.	[27,30,31,40]
Barriers to AI adoption in diabetes care			
The trust deficit: accuracy, bias, and data security			
Concerns regarding algorithmic accuracy, data bias, and unauthorized third-party access are reported as potential barriers to adoption.	High confidence	This is the most richly supported finding (11 studies). Minor methodological limitations did not alter the strong overall coherence.	[27-29,32-36,38-40]
The irreplaceable human element and professional autonomy			
Stakeholders feared that AI could erode clinical empathy, critical thinking, professional autonomy, and patient-clinician rapport.	High confidence	There is a clear consensus on the irreplaceable nature of human touch, offsetting minor concerns in methodological reporting.	[28,29,31,33-36,40]
Increased systemic and caregiver burden			
Anticipated burdens include alert fatigue, data logging, digital exclusion, and caregiver responsibility.	High confidence	Findings are directly relevant to practical implementation barriers across various contexts, with rich data offsetting minor methodological concerns.	[27,28,31,33]
Technical and educational requirements for successful implementation			
Transparency and regulatory compliance			
Clinical validation and understandable explanations are requested to address black-box concerns.	Moderate confidence	Confidence was downgraded to moderate due to moderate concerns regarding adequacy; the finding is coherent but supported by a limited number of studies	[28,29]
Usability and technical preferences			
Stakeholders preferred intuitive, visually oriented interfaces and integration with continuous glucose monitoring and wearable devices.	High confidence	Consistent across 12 primary studies. Minor methodological limitations did not reduce overall adequacy.	[27-29,31-37,39,40]
Segmented education and multichannel support			
Hands-on initial training followed by accessible, ongoing technical and human support is advocated.	High confidence	High coherence across stakeholders regarding training needs offsets minor reflexivity concerns.	[27,28,31-33]

a GRADE-CERQual: Grading of Recommendations Assessment, Development, and Evaluation–Confidence in the Evidence From Reviews of Qualitative Research.

Two reviewers (HM-M and JM-A) independently assessed methodological limitations [43], coherence [44], data adequacy [45], and relevance [46] for the body of evidence contributing to each finding, with disagreements resolved by consensus and, when necessary, consultation with a third author (VM-V). The JBI and CAMELOT assessments were used to inform, rather than mechanically determine, the methodological limitations component of GRADE-CERQual. Concerns were considered at the level of each synthesized finding according to which studies contributed data to that finding. The absence of reported researcher positioning or reflexivity was treated as a recurrent limitation across the evidence base. Ratings reflected the richness, quantity, and distribution of contributing data rather than sample size alone. Potential concerns related to sampling, demographic imbalance, transferability, or exposure to hypothetical or prototype technologies were considered within the methodological limitations, adequacy, or relevance components, as appropriate.

## Results

### Overview

**Figure 1.**
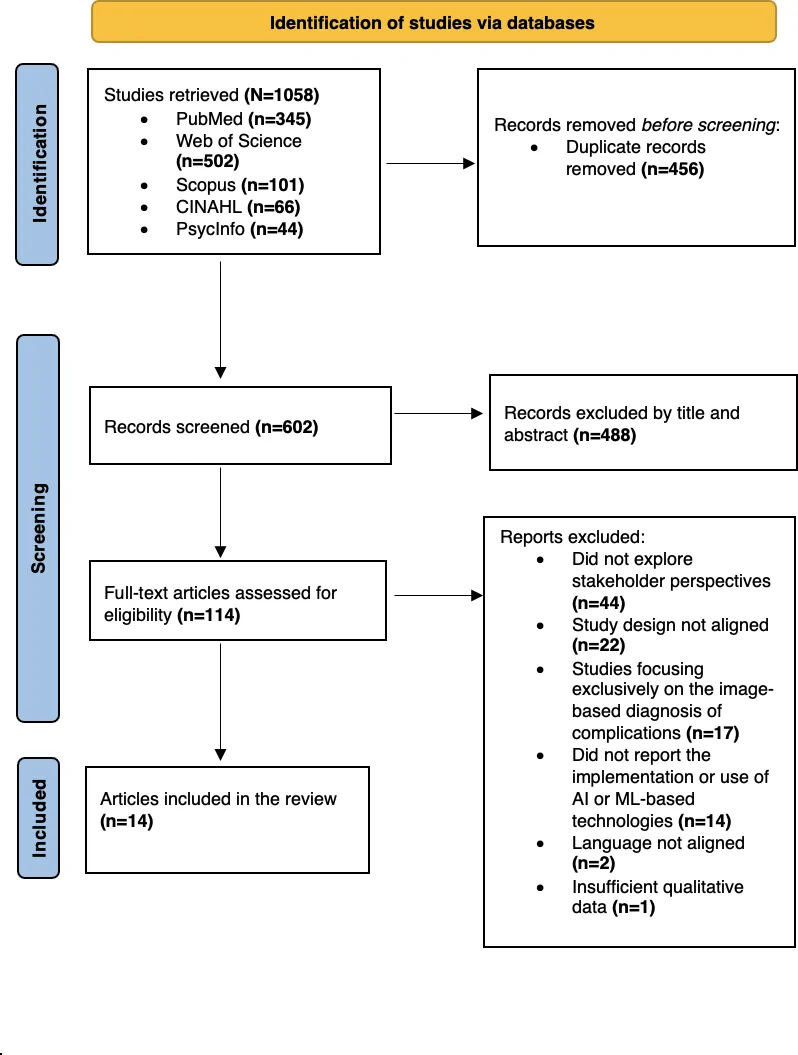
PRISMA (preferred reporting items for systematic reviews and meta-analyses) 2020 flow diagram.ML: machine learning.

After removing duplicates, 602 records underwent title and abstract screening. Of these, 114 articles were selected for full-text review, and 14 [27-40] met the inclusion criteria (see Figure 1). These articles corresponded to 14 unique study populations.

### Characteristics of the Included Studies

A total of 14 studies published between 2023 and 2025 were included in this review (Table 2) [27-40]. Three included studies were conducted in Singapore [27,31,34], 2 in the United States [28,32], 2 in Saudi Arabia [30,39], 2 in Malaysia [33,35], and 1 each in the United Arab Emirates [29], Belgium [36], Germany [37], the United Kingdom [38], and India [40]. Of these, 13 explicitly reported the number of participants [27-39], while one study did not specify the total number of interviewees [40]. Among the studies with available data, the total number of participants was 738. This sample comprised 598 patients with diabetes [27,30-32,35,37-39] and 140 other stakeholders, including health care professionals, hospital staff, and family members [27-29,32-34,36,40]. Participants’ ages ranged from 18 to 81 years, and the overall proportion of female participants was 41.9%.

With respect to data collection, most studies used semistructured interviews [27-29,31,33,35,37,40], focus groups [34], or a combination of interviews and focus groups [32]. Other designs included longitudinal co-design [38], a one-week direct-use study followed by interviews [30], an interactive prototype user study with open-ended feedback [36], and a structured questionnaire containing open-ended responses [39]. Study [39] contributed 418 of the 738 participants with known sample sizes; however, its large numerical sample was not interpreted as providing proportionally greater qualitative weight because its open-ended responses offered less contextual depth than interviews, focus groups, and longitudinal co-design.

The included literature was heterogeneous across stakeholder groups, diabetes types, AI functions, care settings, and levels of exposure (Multimedia Appendix 5). Direct-use studies tended to provide practical accounts of workflow fit, self-management support, and interaction burden, whereas prototype, wireframe, and hypothetical-system studies more often elicited expectations concerning desired features, explainability, trust, and anticipated risks. These patterns were interpreted descriptively because the number and design of studies within each stratum did not support formal comparative subgroup analysis. AI was therefore not treated as a single homogeneous intervention.

### Methodological Quality and Risk of Bias Assessment

Methodological quality was assessed using CAMELOT and JBI Critical Appraisal Checklist for Qualitative Research.

#### CAMELOT Assessment

The included studies were generally methodologically appropriate for their stated qualitative aims, but important limitations were present. Four studies were categorized by CAMELOT as having excellent fit in both design and conduct [27,28,37,40]. Transferability or selection concerns were identified in 5 studies [31,35,36,38,39], including skewed gender distributions [31,35,36,38] and convenience recruitment of predominantly highly educated, technologically literate participants [39]. These concerns were carried forward into the GRADE-CERQual assessment rather than being obscured by an overall study-level label (Multimedia Appendix 4).

#### JBI Assessment

The JBI assessment (Table 1) showed high compliance with qualitative reporting standards across all 14 included studies [27-40]. Congruity between the research methodology and the methods used for data collection and analysis was clearly established in all studies (Items 1‐5). Interpretations and conclusions were consistently supported by the qualitative data (Items 8 and 10), and ethical approval was explicitly reported in all cases (Item 9). However, all 14 studies were rated unclear for JBI items 6 and 7 because researcher cultural or theoretical positioning and the potential influence of the researcher on the research process were insufficiently described.

### Thematic Synthesis

**Figure 2.**
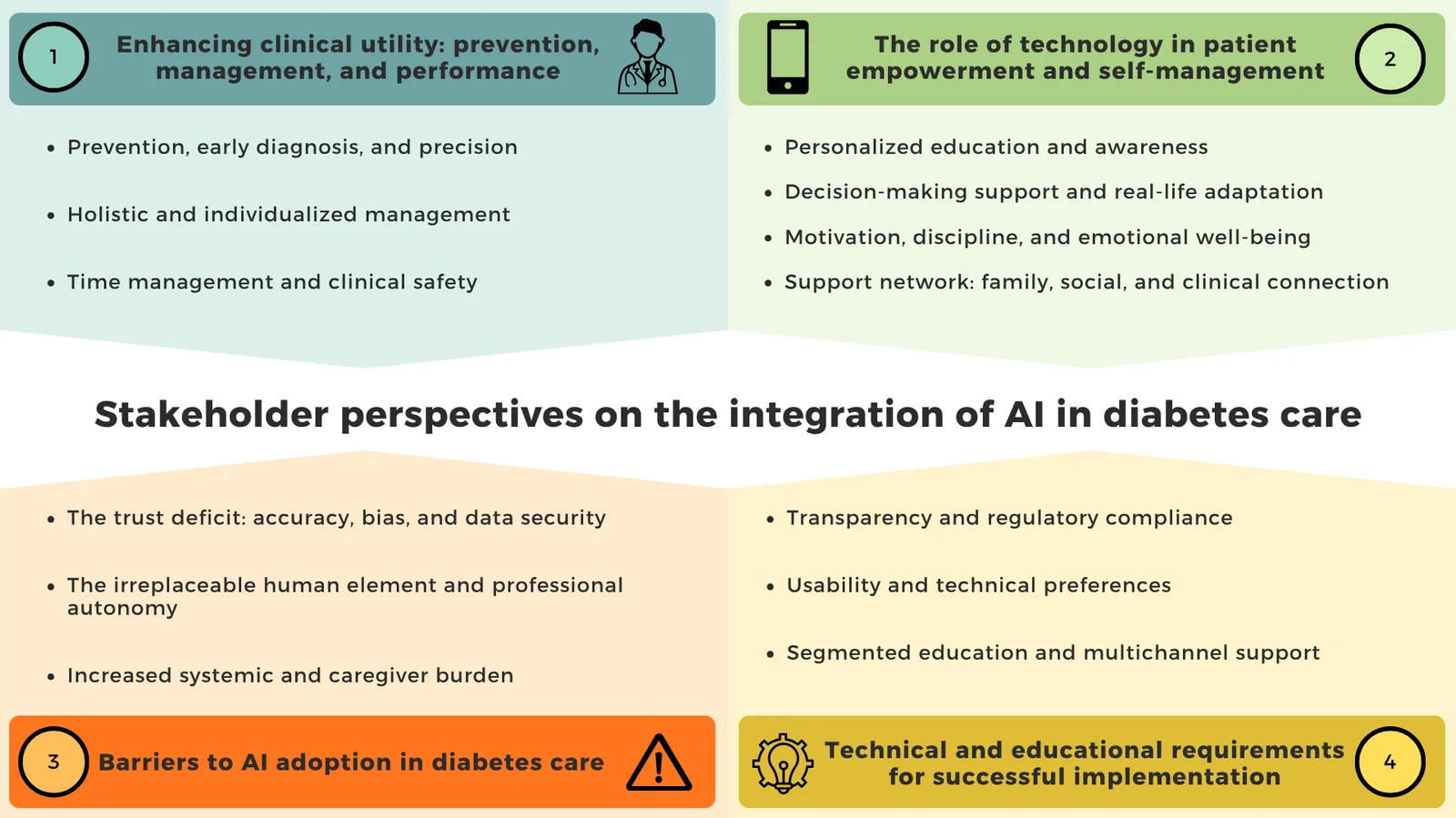
Graphical representation of the themes and subthemes.

A thematic synthesis of the 14 included studies [27-40] revealed 4 main themes and 13 subthemes related to stakeholder perspectives on the integration of AI in diabetes care (Figure 2). All relevant quotations are provided in the supporting information (Multimedia Appendix 7), organized systematically by themes and their corresponding subthemes.

Contributions differed across stakeholder and diabetes groups. Patients most frequently contributed to findings concerning self-management, emotional and practical burden, usability, and access to human support. Health care professionals more often emphasized preventive utility, workflow integration, accuracy, professional autonomy, accountability, and explainability. Family caregivers primarily contributed to shared monitoring, communication, and caregiver burden. Type 1 diabetes mellitus (T1DM)–focused studies particularly addressed glucose variability, insulin decisions, unexpected situations, and device integration, whereas type 2 diabetes mellitus (T2DM)–focused studies more often addressed behavioral support, education, family involvement, risk prediction, and prescribing. The study-level distribution of these contributions is presented in Multimedia Appendix 5.

The sensitivity analysis excluding the questionnaire-based study [39] did not remove or materially alter any of the 4 themes or 13 subthemes. That study contributed supporting data to decision-making support and real-life adaptation, trust and data security, and usability and technical preferences, but none of these findings depended primarily on its data. The GRADE-CERQual confidence ratings were unchanged after its exclusion.

### Theme 1: Enhancing Clinical Utility: Prevention, Management, and Performance

#### Prevention, Early Diagnosis, and Diagnostic Precision

HCPs perceived AI as a potentially valuable tool for shifting diabetes management from reactive treatment toward preventive and anticipatory management. They anticipated that AI could help identify health problems before they worsened and support earlier recognition of diabetes-related complications. AI was also regarded as potentially helpful for recognizing clinical patterns and abnormalities in diagnostic images that might otherwise be overlooked. Clinicians working in fast-paced care settings described AI as a possible additional safety mechanism, and less experienced professionals viewed it as a potential reminder to perform necessary checks, such as eye and foot examinations.

*...the heart of what we do in primary care is to try to help patients with chronic conditions avoid long term complications of those conditions...if [AI believes] this person might be at greater risk, I might see [that patient] more often. I might spend more time with them. I might ask different questions because I would be trying to prevent [the complication]*.

[Family physician] [28]

#### Holistic and Individualized Patient Management

Beyond early diagnosis, AI was viewed as supporting a more comprehensive and individualized approach to care by enabling more frequent monitoring, particularly for patients with limited contact with health care services. Participants perceived these tools as capable of facilitating personalized medicine through the analysis of patient-specific data and the generation of tailored recommendations. Clinicians also anticipated that AI might prompt consideration of factors beyond glycemic measures such as glycated hemoglobin (HbA_1c_), including mental health and social determinants of health. This broader perspective was considered especially relevant for older adults and people living in underserved areas.

*AI helps us personalize treatment plans for each patient. We can predict complications and provide remote monitoring and telemedicine services. This has made managing diabetes much more efficient and effective*.

[HCP] [40]

#### Time Management and Clinical Safety

Participants perceived that AI could support treatment efficiency and clinical safety. In overcrowded, busy clinics, participants felt that AI could reduce congestion and waiting times through remote monitoring, reinforce education between visits, and quickly summarize complex data to support faster clinical decisions.

*Education is crucial in diabetes management. At the clinic, we have limited time for education. Patients often don’t retain information from consultations, so it’s good to have something outside the clinics*.

[Family physician] [33]

### Theme 2: The Role of Technology in Patient Empowerment and Self-Management

#### Personalized Education and Awareness

Users viewed AI as a personalized educational tool that provides tailored diet and exercise advice, guides them toward reliable information, and increases awareness of the long-term impact of self-care behaviors.

*It is a very good powerful supporting tool in diabetes education, especially for patients who can’t access a diabetes educator or DMTAC pharmacist. For hospitals without these services, dedicated patients can find support through these mobile apps. It is very convenient*.

[Diabetes educator] [33]

#### Decision-Making Support and Real-Life Adaptation

Participants reported or anticipated that access to summarized “report cards” and real-time feedback could help users adjust diet, activity, and insulin, while AI might offer context-specific support during illness, holidays, exercise, and other unexpected glucose changes. In these out-of-routine situations, participants viewed technology as a contextualized safety net that could help users reassess unexpected glucose fluctuations.

*I logged my blood sugar levels daily and asked ChatGPT if I needed to change anything. It suggested increasing fiber intake and monitoring for another few days before seeing my doctor. This helped me make informed decisions*.

[Patient] [30]

*It will make patients take ownership of their own self-management, likely making it more effective*.

[Endocrinologist] [33]

#### Motivation, Discipline, and Emotional Well-Being

AI was perceived as a constant source of support that helped patients overcome mental barriers, maintain discipline, reduce emotional stress, and sustain behavior change through goal setting and motivational reinforcement.

*MI would be good to overcome mental barriers. MI can serve as a check-in mechanism to remind me of my progress and how to improve [my behavior]. So even when I am tired, I will still make an effort to exercise*.

[Patient] [31]

#### Support Network: Family, Social, and Clinical Connections

These technologies were seen as strengthening family, social, and clinical support by enabling caregiver monitoring, connecting users to peer groups, and helping patients attend consultations better prepared with questions and digital records.

*With this app, I can monitor a bit more because without it, I don’t really know about the diet and physical activity record. The medication, most of the time, I don’t actively monitor. It’s just that I know that she’s regular about it. If she misses, sometimes she’ll tell me but if she never says, then I don’t know. With the app, it can tell me more, I can play a more active role*.

[Family member] [27]

### Theme 3: Barriers to AI Adoption in Diabetes Care

#### The Trust Deficit: Accuracy, Bias, and Data Security

Trust is essential for adoption, as users value these tools only when outputs are reliable. Key concerns include accuracy, false alerts, clinical misinterpretation, dataset bias, and data security, as well as fears of breaches, insurer access, and unclear responsibility for protecting sensitive information.

*We can use AI but cannot completely because we scared sometime the result or diagnosis from AI will be wrong or some errors. Especially in medical diagnosis, worrying about that*.

[Patient] [35]

*AI systems may be vulnerable to data breaches, posing a risk to patient data and privacy*.

[Endocrinologist or diabetologist] [29]

#### The Irreplaceable Human Element and Professional Autonomy

There is concern that AI could undermine the empathetic, humanistic core of medical care. Behavioral change often depends on personal rapport and emotional support, such as a “reassuring touch,” which algorithms cannot replicate. Physicians also worry that overreliance on AI may weaken critical thinking. When AI recommendations conflict with clinical judgment, specialists tend to rely on their own expertise, as responsibility and liability ultimately remain with the human practitioner.

*I mean the kind of personal touch in MI must be done face-to-face. And even in counseling, I believe sometimes tapping on the shoulder, saying something softly, could change the mood as well*.

[Patient] [31]

*I would say that I’m as good or even better than the system. I don’t feel the need to rely on it; I’ll just do what I do... At the end of the day, we bear the responsibility for our patients...*

[Endocrinologist] [34]

#### Increased Systemic and Caregiver Burden

Participants reported that poorly integrated systems could increase workload and delay consultations, while continuous data logging could also burden patients and caregivers. They identified access barriers that also remain for older adults with limited digital literacy and for rural populations with poor connectivity.

*Say...I have...10 patients in the morning, and all of them have this alert, and so for all of them, I’m taking...these extra steps to identify barriers...that’s going to take more of my time*.

[Family physician] [28]

*If family is going to be involved, it’s a burden and if things don’t move the way they are supposed to move, then I’ll feel like I’m not doing my job as a caregiver*.

[Patient] [27]

### Theme 4: Technical and Educational Requirements for Successful Implementation

#### Transparency and Regulatory Compliance

Participants expressed reluctance toward black-box AI and wanted clear explanations of how risk scores and recommendations were generated. They also regarded regulatory compliance as essential and sought assurance that AI would operate within legal standards for informed consent, accountability, and liability protection.

*The lack of transparency may raise ethical questions and create legal challenges when justifying decisions made using AI*.

[Endocrinologist or diabetologist] [29]

#### Usability and Technical Preferences

Participants indicated that AI tools should be intuitive, with simple navigation and customizable alert frequency that does not disrupt face-to-face interactions. They preferred clear visual elements, such as color-coded recommendations and graphical summaries, rather than dense text. They also viewed integration with smartwatches and existing continuous glucose monitoring (CGM) systems as important for automating data entry and reducing user fatigue.

*Another thing would be making sure that it’s the right time. So again, if I’m in room with the patient, personally, I don’t want to see these pop up, because I’m probably goal-oriented at that moment where I’m trying to put in something specific and this would just slow me down*.

[Family physician] [28]

*So, the tool helps to reinforce my decision-making. The color-coded recommendations provide a clear visual indication, prompting me to address any discrepancies that may arise between the tool’s suggestions and my own clinical plan. In this case, I delve into additional clinical histories that the tool does not have access to and elucidate the rationale behind my decisions. This process enhances my confidence and guides better decision-making during the clinical visit, which can improve the quality of patient care*.

[Endocrinologist] [34]

#### Segmented Education and Multichannel Support

Users advocate for a stepwise learning approach, beginning with basic hands-on orientation and followed by accessible digital resources such as interactive videos. Ongoing support is crucial, including 24/7 technical assistance and access to a human physician when AI recommendations seem inappropriate. For families, clear guidance is needed to help reduce caregiver burden.

*I feel like there should be an option that says contact your doctor or whatever, if it gets to a point where it’s just, the person just feels a little bit off, they’re not agreeing with what’s being advised and everything. There should be an option for them or some pop-up or something that says contact your local physician or something like that*.

[Patient] [32]

#### Assessment of Confidence

Confidence in the 13 review findings was reassessed using GRADE-CERQual (Multimedia Appendix 6). The JBI reflexivity concerns and the CAMELOT concerns regarding selection, transferability, and design fit were mapped to the methodological limitations component for each finding according to the studies contributing to that finding. Adequacy was determined by data richness and distribution across studies rather than total participant counts, while relevance considered stakeholder group, diabetes context, AI function, and whether exposure was direct, prototype-based, or hypothetical. Following assessment, 11 findings were rated as high confidence and 2 as moderate. These ratings refer to the representation of stakeholder perspectives rather than clinical effectiveness.

The universal lack of clear reflexivity reporting was treated as a methodological concern, but generally as a minor rather than serious concern because the synthesized perceptions were supported by participant quotations and recurred across independently conducted studies. Demographic imbalance and transferability concerns led to additional minor concerns in affected studies. High confidence was only retained when these limitations were deemed unlikely to substantially alter a coherent finding supported by sufficiently rich data from multiple contexts.

Variation in technology exposure was also examined. Prototype- and hypothetical-system studies were considered relevant to findings formulated as expectations, preferences, or anticipated barriers, while experience-based interpretations were supported by studies involving direct use of functioning tools, where available. Two subthemes were downgraded to moderate confidence because their concerns were more substantial. Subtheme 2.3 (motivation, discipline, and emotional well-being) had minor coherence concerns and moderate adequacy concerns, as the supporting data were less comprehensive than those for the more technical domains. Subtheme 4.1 (transparency and regulatory compliance) was downgraded due to moderate adequacy concerns, as it was supported by only 2 primary studies, which limited the depth and breadth of the evidence.

## Discussion

### Principal Findings

This systematic review of 14 qualitative studies [27-40] revealed ambivalent but predominantly positive stakeholder perceptions of AI in diabetes care. Supported largely by high-confidence GRADE-CERQual assessments, the findings suggest that patients and HCPs view AI not only as a diagnostic aid, but also as a potentially useful educational and motivational tool. A frequently perceived benefit was its capacity to support a shift from reactive treatment to preventive care [28,33,40]. Notably, this broader perspective was considered especially relevant for older adults and people living in underserved areas, where AI may help bridge critical gaps in health care access [47].

However, participants perceived adoption as limited by a persistent lack of trust, concerns over data privacy, and the potential for increased systemic and caregiver burden. Specifically, the continuous nature of diabetes monitoring led participants to anticipate alert fatigue, data logging burnout, and heightened anxiety when recommendations were overwhelming or lacked clinical accompaniment [27,28,31,33]. Fears that AI could undermine professional autonomy and the essential human touch in care were also prominent [29,34,36]. Concerns regarding algorithmic accuracy are consistent with findings from other domains of digital health, where the “black box” nature of AI triggers institutional skepticism [48]. Nevertheless, this review reveals nuances specific to diabetes care. Participants valued AI as a potential safety net during out-of-routine situations, where usual self-management strategies may fail [38]. While AI applications in other chronic pathologies often emphasize medication adherence [49]**,** participants described diabetes care as requiring lifestyle personalization and management of a substantial mental load [30,31].

Unlike previous quantitative studies that focused mainly on clinicians’ perspectives [50], our findings incorporate the views of patients and caregivers. One notable contribution is the perception of AI as a family communication bridge [27]**,** which underscores the importance of the social and relational functions of digital health tools [51]. This shifts the focus beyond individual monitoring toward supporting the wider network involved in diabetes care. Recent perspectives highlight that large language models (LLMs) offer significant potential for this type of personalized health coaching and nutritional guidance, yet they require rigorous validation and specialized fine-tuning to mitigate critical risks such as algorithmic hallucinations [52].

Stakeholders’ concerns about reliability, privacy, transparency, and cross-institutional deployment also have technical foundations. Multicenter modeling research illustrates efforts to address small samples, distribution differences between sites, knowledge calibration, privacy preservation, generalization, and interpretable classification [53,54]. These studies offer technical context for why participants’ concerns about representative data, explainability, and reliable performance across clinical settings are relevant to implementation.

The primary qualitative evidence synthesized in this review supports that stakeholders prioritize specific technical and systemic requirements for AI adoption. Findings derived directly from the included studies emphasize that usability must prioritize seamless integration with continuous monitoring systems and wearable devices to minimize the burden of manual data entry and mitigate logging fatigue [37,39,40]. Furthermore, stakeholders explicitly demanded transparency, explainability mechanisms, and tailored, multichannel education models to support equitable implementation, particularly for older adults and individuals with lower digital literacy [28,32,33,36].

Broader requirements concerning regulatory classification, formal liability allocation, and institutional governance were not directly evaluated in most of the qualitative studies included. Our interpretation of these issues is informed by external policy frameworks, specifically the World Health Organization (WHO) guidance on AI for health and the European Union Artificial Intelligence Act (EU AI Act) [55,56]. These documents provide the necessary regulatory context regarding transparency, intelligibility, accountability, autonomy, and the protection of sensitive data for high-risk health care systems.

### Strengths and Limitations

A key strength of this review is its multistakeholder approach, which involves the synthesis of perspectives not only from HCPs but also from patients, caregivers, and administrators. By integrating these diverse viewpoints, this review provides a more comprehensive understanding of the sociotechnical factors that shape the implementation of AI in diabetes care. Furthermore, the use of the JBI, CAMELOT, and GRADE-CERQual frameworks enhances the methodological rigor of the synthesis, enabling qualitative findings to be interpreted with high transparency and to inform implementation considerations, clinical practice discussions, or institutional policy development.

However, several limitations must be acknowledged. First, the methodological quality of the primary literature presented specific constraints. In particular, the JBI assessment revealed limited reporting of researcher reflexivity in several studies. This restricts the ability to fully evaluate how researcher positioning, professional backgrounds, and prior assumptions may have influenced data collection and thematic interpretation. Second, potential selection bias was identified in the underlying literature, particularly concerning gender imbalances [31,35,36,38] and overrepresentation of highly educated participants [39]. These demographic patterns may have influenced the findings by overrepresenting individuals who are more familiar with, confident in, or receptive to digital health technologies. Consequently, this review may reflect a more favorable perception of AI acceptability than would be found among populations with lower digital literacy, limited access to technology, or greater socioeconomic vulnerability. Third, the inclusion of only English and Spanish language publications may have excluded relevant studies from other linguistic, cultural, and health care contexts. This limitation could be particularly relevant because attitudes toward AI, data sharing, clinical authority, and self-management technologies are likely to vary across health systems and sociocultural settings. Fourth, it is important to note the nature of the synthesized evidence. These qualitative findings describe stakeholder experiences, expectations, and beliefs; they do not demonstrate clinical effectiveness, safety, diagnostic accuracy, or improved patient outcomes, which must be established through quantitative and experimental designs. Fifth, the evidence base was heterogeneous across stakeholder groups, diabetes types, AI modalities, care settings, and level of exposure. Some participants described direct experience with functioning tools, while others evaluated prototypes, wireframes, general concepts, or hypothetical systems. Perceptions based on anticipated use may not predict responses after sustained real-world exposure, and the synthesis should therefore not be read as a uniform estimate of acceptability across technologies or populations. Finally, because conversational LLMs and associated technologies are evolving rapidly, stakeholder perceptions may shift faster than conventional scientific publication timelines can capture.

### Conclusions

Stakeholders perceived AI as offering possibilities for continuous, personalized support beyond traditional clinical encounters, as well as assisting with decision-making, patient empowerment, and more responsive models of care. However, these findings reflect qualitative perceptions, experiences, and expectations and should not be interpreted as evidence that AI improves clinical effectiveness, safety, or patient outcomes. Successful implementation will depend on addressing concerns regarding trust, transparency, accountability, equity, and the preservation of human-centered care. The future of AI in diabetes care should therefore be guided by transparent design, meaningful stakeholder involvement, and careful integration into existing clinical, social, and family support networks. Rather than replacing human relationships in care, AI should function as a supportive tool that strengthens communication, shared decision-making, and individualized diabetes management.

## Supplementary material

10.2196/105329 Multimedia Appendix 1 ENTREQ statement.

10.2196/105329 Multimedia Appendix 2 Search strategy adapted from the SPIDER tool.

10.2196/105329 Multimedia Appendix 3 Search strategy for each database.

10.2196/105329 Multimedia Appendix 4 Summary of the methodological limitations of the included studies (CAMELOT).

10.2196/105329 Multimedia Appendix 5 Study-level evidence map by population, AI function, exposure, and analytical theme.

10.2196/105329 Multimedia Appendix 6 Evidence profile table (GRADE CERQual).

10.2196/105329 Multimedia Appendix 7 Quotations by themes and subthemes.

10.2196/105329 Checklist 1 PRISMA 2020 checklist.

## Abbreviations

CAMELOT: Cochrane Qualitative Methodological Limitations Tool
CGM: continuous glucose monitoring
DM: diabetes mellitus
ENTREQ: Enhancing Transparency in Reporting the Synthesis of Qualitative Research
EU AI Act: European Union Artificial Intelligence Act
GRADE-CERQual: Grading of Recommendations Assessment, Development and Evaluation Confidence in the Evidence from Reviews of Qualitative Research
HbA_1c_: glycated hemoglobin
HCP: health care professional
JBI: Joanna Briggs Institute
LLM: large language model
mHealth: mobile health
ML: Machine Learning
PROSPERO: International Prospective Register of Systematic Reviews
SPIDER: sample, phenomenon of interest, design, evaluation, and research type
WHO: World Health Organization
