# Supplementary material for: Stakeholder Perspectives on the Integration of AI in Diabetes Care: Systematic Review of Qualitative Studies
Source: J Med Internet Res. 2026 Sep 22;28:e105329. doi: 10.2196/105329 (PMC13596760; doi:10.2196/105329)
Supplement: Supplementary file 3 [file jmir-v28-e105329-s002.docx]

**Search strategy adapted from the sample, phenomenon of interest, design, evaluation, and research type (SPIDER) tool**

| **Sample** | • **Inclusion:** Adults (≥18) with diabetes mellitus (T1DM, T2DM), family caregivers, and/or healthcare professionals (HCPs) involved in diabetes care.  • **Exclusion:** Studies conducted exclusively in pediatric populations. Studies where the sample consists exclusively of non-clinical stakeholders (e.g., software developers with no clinical contact). |
| --- | --- |
| **Phenomenon of interest** | • **Inclusion:** The views, perceptions, and experiences regarding the use, acceptance, and implementation of AI tools for diabetes management, Glc control, and risk prevention.  • **Exclusion:** Digital health technologies (telehealth, mobile apps) that do not explicitly incorporate AI components or predictive algorithms. |
| **Design** | • **Inclusion:** Qualitative research designs, such as ethnography, phenomenology, case studies, and grounded theory, that use qualitative data collection methods (e.g., interviews, focus groups).  • **Exclusion:** Mixed-methods studies will be included only if the qualitative data and findings are reported and can be analyzed separately. |
| **Evaluation** | • **Inclusion:** The direct experiences, perceptions, attitudes, and concerns of patients, caregivers, or HCPs regarding the adoption of AI in clinical or home settings.  • **Exclusion:** Evaluations focused only on user interface satisfaction or usability metrics without exploring the underlying perception of the technology or its impact on disease management. |
| **Research type** | • **Inclusion:** Published, peer-reviewed primary qualitative research.  • **Exclusion:** Studies focusing only on technical performance, algorithm development, or validation of AI models without exploring user perceptions. Quantitative studies, study protocols, systematic reviews, doctoral theses, and gray literature. |

**Abbreviations:** AI: artificial intelligence; Glc: glucose; DKA: diabetic ketoacidosis; HCPs: healthcare professionals; T1DM: type 1 diabetes mellitus; T2DM: type 2 diabetes mellitus.
