# Supplementary material for: Stakeholder Perspectives on the Integration of AI in Diabetes Care: Systematic Review of Qualitative Studies
Source: J Med Internet Res. 2026 Sep 22;28:e105329. doi: 10.2196/105329 (PMC13596760; doi:10.2196/105329)
Supplement: Supplementary file 4 [file jmir-v28-e105329-s003.docx]

**Search strategy for each database**

**PUBMED**

| **S1** | "Diabetes Mellitus"[MeSH] OR Diabet*[Title/Abstract] OR "Prediabetic State"[MeSH] OR Prediabet*[Title/Abstract] OR "Glycemic Control"[Title/Abstract] OR "Blood Glucose Self-Monitoring"[MeSH] OR "Blood Glucose Self-Monitoring"[Title/Abstract] OR "Hypoglycemia"[MeSH] OR Hypoglycem*[Title/Abstract] OR "Continuous Glucose Monitoring"[Title/Abstract] OR CGM[Title/Abstract] OR "Insulin Infusion Systems"[MeSH] OR "Insulin Pump*"[Title/Abstract] OR "Diabetic Ketoacidosis"[MeSH] OR "Diabetic Ketoacidosis"[Title/Abstract] |
| --- | --- |
| **S2** | "Artificial Intelligence"[Mesh] OR "Artificial Intelligence"[Title/Abstract] OR AI[Title/Abstract] OR "Machine Learning"[Mesh] OR "Machine Learning"[Title/Abstract] OR "Deep Learning"[Mesh] OR "Deep Learning"[Title/Abstract] OR "Neural Networks, Computer"[Mesh] OR "Neural Network*"[Title/Abstract] OR "Natural Language Processing"[Mesh] OR "Natural Language Processing"[Title/Abstract] OR "Large Language Models"[Mesh] OR "Large Language Model*"[Title/Abstract] OR "Computer-Aided Detection"[Title/Abstract] OR "Computer-Aided Diagnosis"[Title/Abstract] OR "Decision Support Systems, Clinical"[Mesh] OR "Decision Support System*”[Title/Abstract] OR "Clinical Decision Support"[Title/Abstract] |
| **S3** | "Qualitative Research"[Mesh] OR Qualitative[Title/Abstract] OR "Mixed Methods"[Title/Abstract] OR "Mixed-Methods"[Title/Abstract] OR "Interviews as Topic"[Mesh] OR Interview*[Title/Abstract] OR "Semi-structured Interview*"[Title/Abstract] OR "Focus Groups"[Mesh] OR "Focus Group*"[Title/Abstract] OR "Grounded Theory"[Title/Abstract] OR "Thematic Analysis"[Title/Abstract] OR "Content Analysis"[Title/Abstract] OR "Narrative Research"[Title/Abstract] OR "Phenomenological Research"[Title/Abstract] |
| **S4** | Perception[Mesh] OR Perception*[Title/Abstract] OR Attitude[Mesh] OR Attitude*[Title/Abstract] OR "Health Knowledge, Attitudes, Practice"[Mesh] OR "Patient Satisfaction"[Mesh] OR Satisfaction[Title/Abstract] OR Fear[Mesh] OR Fear*[Title/Abstract] OR Uncertainty[Mesh] OR Uncertaint*[Title/Abstract] OR Trust[Mesh] OR Trust*[Title/Abstract] OR Perspective*[Title/Abstract] OR View[Title/Abstract] OR Opinion*[Title/Abstract] OR Experience*[Title/Abstract] OR Concern*[Title/Abstract] OR Worr*[Title/Abstract] OR Expectation*[Title/Abstract] OR Priorit*[Title/Abstract] OR Value[Title/Abstract] OR Confidence*[Title/Abstract] OR Reliabilit*[Title/Abstract] OR Dependabilit*[Title/Abstract] OR Understanding*[Title/Abstract] OR Implication*[Title/Abstract] OR Enabler*[Title/Abstract] OR Barrier*[Title/Abstract] OR Accept*[Title/Abstract] OR Adoption*[Title/Abstract] OR "Willingness to Use"[Title/Abstract] OR Resistance*[Title/Abstract] OR Feasibilit*[Title/Abstract] OR Implementation*[Title/Abstract] OR Adoptabilit*[Title/Abstract] OR perceive*[Title/Abstract] OR Motivation[Mesh] OR Motivation*[Title/Abstract] |
| **S5** | S1 AND S2 AND S3 AND S4 |

**Web of Science**

| **S1** | AB=(Diabet* OR Prediabet* OR "Glycemic Control" OR "Blood Glucose Self-Monitoring" OR Hypoglycem* OR "Continuous Glucose Monitoring" OR CGM OR "Insulin Infusion Systems" OR "Insulin Pump*" OR "Diabetic Ketoacidosis") |
| --- | --- |
| **S2** | AB=("Artificial Intelligence" OR AI OR "Machine Learning" OR "Deep Learning" OR "Neural Network*" OR "Natural Language Processing" OR "Large Language Model*" OR "Computer-Aided Detection" OR "Computer-Aided Diagnosis" OR "Decision Support System*" OR "Clinical Decision Support") |
| **S3** | AB=("Qualitative Research" OR "Mixed Method*" OR "Mixed-Method*" OR Interview* OR "Focus Group*" OR "Grounded Theory" OR "Thematic Analysis" OR "Content Analysis" OR "Narrative Research" OR "Phenomenological Research" OR qualitative OR "semi-structured interview*") |
| **S4** | AB=(Perception* OR Attitude* OR Satisfaction OR Perspective* OR Opinion* OR Experience* OR View* OR Concern* OR Worr* OR Fear* OR Uncertaint* OR Expectation* OR Value* OR Trust* OR Confidence* OR Reliabilit* OR Dependabilit* OR Understanding* OR Implication* OR Priorit* OR Enabler* OR Barrier* OR Accept* OR Adoption* OR "Willingness to Use" OR Resistance* OR Feasibilit* OR Implementation*OR Adoptabilit* OR Perceive* OR Motivation*) |
| **S5** | S1 AND S2 AND S3 AND S4 |

**SCOPUS**

| **S1** | TITLE-ABS-KEY((Diabet* OR Prediabet* OR "Glycemic Control" OR "Blood Glucose Self-Monitoring" OR Hypoglycem* OR "Continuous Glucose Monitoring" OR CGM OR "Insulin Infusion Systems" OR "Insulin Pump*" OR "Diabetic Ketoacidosis")) |
| --- | --- |
| **S2** | TITLE-ABS-KEY(("Artificial Intelligence" OR AI OR "Machine Learning" OR "Deep Learning" OR "Neural Network*" OR "Natural Language Processing" OR "Large Language Model*" OR "Computer-Aided Detection" OR "Computer-Aided Diagnosis" OR "Decision Support System*" OR "Clinical Decision Support")) |
| **S3** | TITLE-ABS-KEY(("Qualitative Research" OR "Mixed Method*" OR "Mixed-Method*” OR Interview* OR "Focus Group*" OR "Grounded Theory" OR "Thematic Analysis" OR "Content Analysis" OR "Narrative Research" OR "Phenomenological Research" OR qualitative OR "semi-structured interview*")) |
| **S4** | TITLE-ABS-KEY((Perception* OR Attitude* OR Satisfaction OR Perspective* OR Opinion* OR Experience* OR View* OR Concern* OR Worr* OR Fear* OR Uncertaint* OR Expectation* OR Value* OR Trust* OR Confidence* OR Reliabilit* OR Dependabilit* OR Understanding* OR Implication* OR Priorit* OR Enabler* OR Barrier* OR Accept* OR Adoption* OR "Willingness to Use" OR Resistance* OR Feasibilit* OR Implementation*OR Adoptabilit* OR Perceive* OR Motivation*)) |
| **S5** | S1 AND S2 AND S3 AND S4 |

**CINAHL**

| **S1** | XB (Diabet* OR Prediabet* OR "Glycemic Control" OR "Blood Glucose Self-Monitoring" OR Hypoglycem* OR "Continuous Glucose Monitoring" OR CGM OR "Insulin Infusion Systems" OR "Insulin Pump*" OR "Diabetic Ketoacidosis") |
| --- | --- |
| **S2** | XB ("Artificial Intelligence" OR AI OR "Machine Learning" OR "Deep Learning" OR "Neural Network*" OR "Natural Language Processing" OR "Large Language Model*" OR "Computer-Aided Detection" OR "Computer-Aided Diagnosis" OR "Decision Support System*" OR "Clinical Decision Support") |
| **S3** | XB ("Qualitative Research" OR "Mixed Method*" OR "Mixed-Method*" OR Interview* OR "Focus Group*" OR "Grounded Theory" OR "Thematic Analysis" OR "Content Analysis" OR "Narrative Research" OR "Phenomenological Research" OR qualitative OR "semi-structured interview*") |
| **S4** | XB (Perception* OR Attitude* OR Satisfaction OR Perspective* OR Opinion* OR Experience* OR View* OR Concern* OR Worr* OR Fear* OR Uncertaint* OR Expectation* OR Value* OR Trust* OR Confidence* OR Reliabilit* OR Dependabilit* OR Understanding* OR Implication* OR Priorit* OR Enabler* OR Barrier* OR Accept* OR Adoption* OR "Willingness to Use" OR Resistance* OR Feasibilit* OR Implementation*OR Adoptabilit* OR Perceive* OR Motivation*) |
| **S5** | S1 AND S2 AND S3 AND S4 |

**APA PsycInfo**

| **S1** | XB (Diabet* OR Prediabet* OR "Glycemic Control" OR "Blood Glucose Self-Monitoring" OR Hypoglycem* OR "Continuous Glucose Monitoring" OR CGM OR "Insulin Infusion Systems" OR "Insulin Pump*" OR "Diabetic Ketoacidosis") |
| --- | --- |
| **S2** | XB ("Artificial Intelligence" OR AI OR "Machine Learning" OR "Deep Learning" OR "Neural Network*" OR "Natural Language Processing" OR "Large Language Model*" OR "Computer-Aided Detection" OR "Computer-Aided Diagnosis" OR "Decision Support System*" OR "Clinical Decision Support") |
| **S3** | XB ("Qualitative Research" OR "Mixed Method*" OR "Mixed-Method*" OR Interview* OR "Focus Group*" OR "Grounded Theory" OR "Thematic Analysis" OR "Content Analysis" OR "Narrative Research" OR "Phenomenological Research" OR qualitative OR "semi-structured interview*") |
| **S4** | XB (Perception* OR Attitude* OR Satisfaction OR Perspective* OR Opinion* OR Experience* OR View* OR Concern* OR Worr* OR Fear* OR Uncertaint* OR Expectation* OR Value* OR Trust* OR Confidence* OR Reliabilit* OR Dependabilit* OR Understanding* OR Implication* OR Priorit* OR Enabler* OR Barrier* OR Accept* OR Adoption* OR "Willingness to Use" OR Resistance* OR Feasibilit* OR Implementation*OR Adoptabilit* OR Perceive* OR Motivation*) |
| **S5** | S1 AND S2 AND S3 AND S4 |
