# Supplementary material for: Stakeholder Perspectives on the Integration of AI in Diabetes Care: Systematic Review of Qualitative Studies
Source: J Med Internet Res. 2026 Sep 22;28:e105329. doi: 10.2196/105329 (PMC13596760; doi:10.2196/105329)
Supplement: Supplementary file 5 [file jmir-v28-e105329-s004.docx]

**. Summary of the methodological limitations of the included studies. Cochrane qualitative methodological limitations tool (CAMELOT).**

| **Study (Author, Year)** | **Study Context (META Domains Summary)** | **Design Assessment (Fit & Rationale)** | **Conduct Assessment (Fit & Rationale)** | **Overall Assessment (Level of Concern & Final Rationale)** |
| --- | --- | --- | --- | --- |
| **Yoon, Sungwon et al.** [33]**, 2025** | Qualitative study on the acceptability of AI-enabled diabetes management in Singapore. Publicly funded; no conflicts of interest declared. | **Excellent.** Integration of mock wireframes into interviews provided a concrete basis for evaluating hypothetical AI functionalities, ensuring alignment with exploratory aims. | **Excellent.** Methodology included independent dual coding and member checking during data collection to ensure data dependability. Reporting follows COREQ guidelines. | **No or minimal concerns.** Methodological procedures demonstrate strong alignment with the study objectives. Wireframe utilization effectively addressed the challenge of evaluating non-implemented technologies. |
| **Liaw, Winston R. et al.** [34]**, 2023** | Mixed-methods study conducted in US safety-net clinics. Academic research team supported by federal funding; no commercial conflicts of interest declared. | **Excellent.** The qualitative design was appropriately integrated to explain low adoption rates observed in the quantitative phase, providing a robust fit with the exploratory objectives. | **Excellent.** Methodology included dual independent coding and assessment of inter-rater reliability. Clear reporting of participant characteristics and thematic derivation. | **No or minimal concerns.** Methodological procedures demonstrate strong alignment with the study aims. The use of purposive sampling effectively captured a range of perspectives from different staff roles. |
| **Jairoun, Ammar Abdulrahman et al.**[35]**, 2024** | Qualitative study exploring specialist perspectives on ChatGPT applications in the UAE. Academic research team; no specific funding or conflicts of interest declared. | **Good.** The semi-structured interview design was an appropriate choice for capturing professional insights into a rapidly evolving generative AI technology, ensuring a good fit with the exploratory aims. | **Good.** Standard thematic analysis was utilized, with data saturation reported. The description of the coding process is adequate, although specific details regarding inter-coder reliability protocols were not reported. | **No or minimal concerns.** Methodological procedures demonstrate alignment with the study objectives. The findings provide relevant evidence on clinical risks and benefits from a specialist perspective. |
| **Alanzi, Turki M. et al.** [36]**, 2025** | Qualitative-experimental study evaluating ChatGPT's impact on DM self-management in Saudi Arabia. Academic research team; no financial support or conflicts of interest declared. | **Good.** The qualitative-experimental design, including a structured interaction period with the AI tool, provided a concrete basis for capturing user perceptions, ensuring alignment with the study aims. | **Good.** Analysis followed a six-step thematic process with independent dual coding. Transcripts were professionally translated. Data saturation was justified within the sample size. | **No or minimal concerns.** Methodological procedures demonstrate alignment with objectives. The use of purposive sampling and a specific interaction period adds reliability to the findings on patient engagement. |
| **Yoon, Sungwon et al.** [37]**, 2024** | Qualitative study on the acceptability of app-based motivational interviewing among T2DM patients in Singapore. Academic research team; no conflicts of interest declared. | **Good.** The use of semi-structured interviews was an appropriate design choice to capture in-depth perspectives on the barriers and facilitators of digital health interventions, ensuring a good fit with the exploratory objectives. | **Good.** Methodological procedures included inductive thematic analysis with dual coding and group consensus. Reporting follows COREQ guidelines. | **Minor concerns.** Methodological procedures demonstrate alignment with study objectives. Recruitment from a previous trial population ensured relevant participant experience with digital health. However, the sample was skewed toward male participants, limiting transferability. |
| **Robinson, Renee et al.** [38]**, 2023** | Qualitative study exploring patient information needs and transparency design for AI in diabetes. Conducted by an academic pharmacy/nursing team and funded by the FDA; no commercial conflicts of interest declared. | **Excellent.** The use of focus groups was appropriate for stimulating discussion on safety and trust, while the use of visual prototypes provided a concrete basis for evaluating transparency needs, ensuring alignment with the study aims. | **Good.** Recruitment achieved a diverse sample of T1DM/T2DM patients and providers. The analytic process followed an iterative coding process and constant comparison within the CFIR and grounded theory frameworks. | **No or minimal concerns.** Recruitment achieved a diverse sample of T1DM/T2DM patients and providers. The analytic process followed an iterative coding process and constant comparison within the CFIR and grounded theory frameworks. |
| **Lim, Phei-Ching et al.** [39]**, 2025** | Qualitative study investigating Malaysian HCPs' perspectives on AI-based apps for diabetes management. Funded by the Ministry of Higher Education; no conflicts of interest declared. | **Good.** The use of semi-structured interviews was appropriate for capturing professional insights from diverse healthcare roles, ensuring alignment with the exploratory aims. | **Good.** The analysis followed established thematic procedures using NVivo 14. Data saturation was reported, and the sample provided a representative range of professional perspectives within the context. | **No or minimal concerns.** Methodological procedures demonstrate alignment with study objectives. The inclusion of participants from multiple specialties and geographical areas in Malaysia enhances the relevance of the findings. |
| **Yoon, Sungwon et al.**[40]**, 2024** | Qualitative study in a tertiary public hospital in Singapore. Academic/clinical research team; funded by institutional/public grants; no commercial conflicts of interest declared. | **Good.** The use of focus groups organized by seniority was an appropriate design choice to capture collective professional norms and hierarchy-related barriers, ensuring alignment with the exploratory aims. | **Good.** Methodology included independent dual coding and reflexive thematic analysis. Data saturation was reported, and the sample provided specific perspectives from specialists in a specialized setting. | **No or minimal concerns.** Methodological procedures demonstrate strong alignment with the research objectives. The focus on a specific professional group provides high-value evidence for clinical decision support adoption. |
| **Krisnan, Logeswary et al.** [41]**, 2025** | Qualitative study investigating patient perceptions of AI in diabetes care in Malaysia. Funded by the Qualitative Research Association of Malaysia; no conflicts of interest declared. | **Good.** The use of semi-structured interviews was appropriate for exploring subjective experiences and attitudes within a public hospital setting, ensuring alignment with the research aim. | **Good.** Analysis followed Braun and Clarke's framework with independent dual coding and consensus resolution. Data saturation was reported, and ATLAS.ti was used for data management. | **Minor concerns.** Methodological procedures demonstrate alignment with study objectives. Although the gender balance is skewed toward females, the study provides relevant insights into patient expectations and trust. |
| **Samimi, Reza et al.**[42]**, 2025** | Mixed-methods study evaluating a DSS prototype for diabetes risk. Academic research team; publicly funded; no commercial conflicts of interest declared. | **Good.** The inclusion of a co-design phase with nurses and the use of specific tasks provided a structured framework for evaluating the visual-conversational interface, ensuring alignment with the evaluative aims. | **Good.** Analysis included independent dual coding with an inter-rater reliability score (Cohen’s Kappa) of 0.81. Recruitment was validated via domain knowledge testing to ensure expert perspectives. | **Minor concerns.** Procedures align with objectives. However, the sample was skewed toward female participants, limiting transferability. |
| **Klemme, Isabel et al.**[43]**, 2023** | Qualitative study exploring patient visions for future diabetes technology in Germany. Funded by the German Research Foundation; no conflicts of interest declared. | **Excellent.** The use of the RRI vision assessment framework provided a systematic method for eliciting and analyzing patient expectations for future AI functionalities, ensuring a strong fit with the exploratory aims. | **Excellent.** Methodological procedures included inductive and deductive coding phases using ATLAS.ti. Data saturation was reported, and the sample provided a broad range of expert perspectives. | **No or minimal concerns.** Methodological procedures demonstrate alignment with the research objectives. The theoretical framework effectively guided the identification of future-oriented technological needs and AI-driven features. |
| **Stawarz, Katarzyna et al.** [44]**, 2023** | Longitudinal co-design study in the UK investigating ML-based support for T1DM. Academic team; funded by Innovate UK; no conflicts of interest declared. | **Excellent.** The 9-month longitudinal design provided a superior basis for differentiating between superficial user preferences and actual self-management needs, ensuring a robust fit with the exploratory goals. | **Good.** Sustained engagement with participants facilitated deep insights. Analysis followed established thematic procedures with team consensus. The sample was heavily skewed toward male participants. | **Minor concerns**. Methodological procedures demonstrate strong alignment with the research objectives. While the gender imbalance limits transferability, the depth and duration of data collection provide high confidence in the specific findings on non-routine decision-making. |
| **Alzghaibi, Haitham,** [45] **2025** | Mixed-methods study investigating perceptions of AI-integrated wearables in Saudi Arabia. Academic affiliation; funded by Qassim University; no conflicts of interest declared. | **Good.** The cross-sectional design, incorporating open-ended questions, provided a suitable framework for identifying broad trends and specific user concerns, ensuring alignment with the exploratory aims. | **Fair.** While thematic analysis was utilized for qualitative data, the reliance on convenience sampling via online platforms introduced a selection bias toward highly educated participants. | **Minor concerns.** Methodological procedures align with objectives. However, findings primarily represent a specific demographic of technologically literate users, limiting the transferability to the broader diabetic population |
| **Roy, Mrinmoy et al.** [46]**, 2025** | Qualitative study investigating the adoption of AI-based diabetes diagnosis in non-metropolitan India. Dual perspective from institutional buyers and end-users. Academic research team; no specific funding declared; no commercial conflicts of interest declared. | **Excellent. T**he qualitative design using semi-structured interviews was highly appropriate for capturing in-depth operational and experiential perspectives from both management and patients. | **Excellent.** Rigorous execution utilizing the six-step thematic analysis. Procedures included pilot validation, investigator triangulation (3 researchers), and formal IRB approval. | **No or minimal concerns.** Methodological procedures demonstrate strong alignment with study objectives. The dual-perspective approach and triangulation provide high confidence in the findings regarding tier-2 and tier-3 cities. |

**Abbreviations:** COREQ: Consolidated Criteria for Reporting Qualitative Research; HCP: Healthcare Professional; SAU: Saudi Arabia; DM: Diabetes Mellitus; T1DM: Type 1 Diabetes Mellitus; T2DM: Type 2 Diabetes Mellitus; CFIR: Consolidated Framework for Implementation Research; FDA: Food and Drug Administration; DSS: Decision Support System; BEL: Belgium; RRI: Responsible Research and Innovation; IRB: Institutional Review Board.

**REFERENCES**

1. 33. Lim PC, Chong YW, Chie QT, Zainal H, Yau KLA, Teoh SH. Healthcare professionals’ perspectives on artificial intelligence (AI)-based mobile applications (apps) for diabetes education and behavioural management. Digit HEALTH. 2025;11:20552076251329991. [doi: ] [Medline: 40162163]
2. 34. Yoon S, Goh H, Lee PC, et al. Assessing the utility, impact, and adoption challenges of an artificial intelligence-enabled prescription advisory tool for type 2 diabetes management: qualitative study. JMIR Hum Factors. Jun 13, 2024;11:e50939. [doi: ] [Medline: 38869934]
3. 35. Krisnan L, Masrom M, Yahya Y, Krishnan M. Patient perceptions toward the application of artificial intelligence in diabetes care: a qualitative study. Asian J Soc Health Behav. 2025;8(1):15-20. [doi: ]
4. 36. Samimi R, Bhattacharya A, Gosak L, Stiglic G, Verbert K. Visual-conversational interface for evidence-based explanation of diabetes risk prediction. In: CUI ’25: Proceedings of the 7th ACM Conference on Conversational User Interfaces. Association for Computing Machinery; 2025. [doi: ]
5. 37. Klemme I, Wrona KJ, de Jong IM, Dockweiler C, Aschentrup L, Albrecht J. Integration of the vision of people with diabetes Into the development process to improve self-management via diabetes apps: qualitative interview study. JMIR Diabetes. Apr 27, 2023;8:e38474. [doi: ] [Medline: 37104003]
6. 38. Stawarz K, Katz D, Ayobi A, et al. Co-designing opportunities for human-centred machine learning in supporting type 1 diabetes decision-making. Int J Hum Comput Stud. May 2023;173:103003. [doi: ]
7. 39. Alzghaibi H. Perspectives of people with diabetes on AI-integrated wearable devices: perceived benefits, barriers, and opportunities for self-management. Front Med (Lausanne). 2025;12. [doi: ] [Medline: 40337275]
8. 40. Roy M, Singh M, Jamwal M. Adoption of AI-based diabetes diagnosis: a patient and institutional perspective in Maharashtra and Karnataka. Int J Pharm Healthc Mark. Apr 30, 2026;20(2):365-383. [doi: ]
9. 41. Thomas J, Harden A. Methods for the thematic synthesis of qualitative research in systematic reviews. BMC Med Res Methodol. Jul 10, 2008;8(1):45. [doi: ] [Medline: 18616818]
10. 42. Lewin S, Bohren M, Rashidian A, et al. Applying GRADE-CERQual to qualitative evidence synthesis findings—paper 2: how to make an overall CERQual assessment of confidence and create a summary of qualitative findings table. Implementation Sci. Jan 2018;13(S1):10. [doi: ]
11. 43. Munthe-Kaas H, Bohren MA, Glenton C, et al. Applying GRADE-CERQual to qualitative evidence synthesis findings—paper 3: how to assess methodological limitations. Implementation Sci. Jan 2018;13(S1):9. [doi: ]
12. 44. Colvin CJ, Garside R, Wainwright M, et al. Applying GRADE-CERQual to qualitative evidence synthesis findings—paper 4: how to assess coherence. Implementation Sci. Jan 2018;13(S1):13. [doi: ]
13. 45. Glenton C, Carlsen B, Lewin S, et al. Applying GRADE-CERQual to qualitative evidence synthesis findings—paper 5: how to assess adequacy of data. Implementation Sci. Jan 2018;13(S1):14. [doi: ]
14. 46. Noyes J, Booth A, Lewin S, et al. Applying GRADE-CERQual to qualitative evidence synthesis findings–paper 6: how to assess relevance of the data. Implementation Sci. Jan 2018;13(S1):4. [doi: ]
