# Supplementary material for: Stakeholder Perspectives on the Integration of AI in Diabetes Care: Systematic Review of Qualitative Studies
Source: J Med Internet Res. 2026 Sep 22;28:e105329. doi: 10.2196/105329 (PMC13596760; doi:10.2196/105329)
Supplement: Supplementary file 6 [file jmir-v28-e105329-s005.docx]

**Multimedia Appendix 5. Study-level evidence map by population, AI function, exposure, and analytical theme**

|  | **Stakeholders** | **Diabetes/context** | **AI function** | **Exposure** | **Perceived clinical utility** | **Empowerment/self-management** | **Adoption barriers** | **Technical/educational requirements** |
| --- | --- | --- | --- | --- | --- | --- | --- | --- |
| [33] | Patients; family caregivers | T2DM; family-supported self-management | AI mHealth nudges and food recognition | Wireframes | Yes | Yes | Yes | Yes |
| [34] | Clinicians and clinical staff | Diabetes risk stratification in primary care | EHR prediction tool | Functioning system | Yes | Yes | Yes | Yes |
| [35] | Endocrinologists; diabetologists | Diabetes and metabolic disease | Generative AI/ChatGPT | Conceptual/professional assessment | Yes | No | Yes | Yes |
| [36] | Patients | Diabetes type not disaggregated; self-management | ChatGPT | Direct use for one week | No | Yes | No | No |
| [37] | Patients | T2DM; behavioral support | Automated motivational interviewing app | Mock-up wireframe | Yes | Yes | Yes | Yes |
| [38] | Patients; HCPs | T1DM and T2DM; glucose prediction/insulin dosing | AI/ML transparency displays | Hypothetical displays | No | No | Yes | Yes |
| [39] | HCPs | Diabetes education and behavioral management | AI-enabled mobile applications | General concept | Yes | Yes | Yes | Yes |
| [40] | Endocrinologists | T2DM; prescribing | AI prescription advisory tool | Functioning/prototype tool | Yes | No | Yes | Yes |
| [41] | Patients | T2DM; primary care | General AI monitoring, diagnosis, and alerts | General concept | Yes | No | Yes | Yes |
| [42] | Nurses, physicians, and other HCPs | Glucose-related risk prediction | Visual-conversational decision support | Interactive prototype | Yes | No | Yes | Yes |
| [43] | Patients | T1DM; apps and wearables | Glucose prediction and AI-supported app functions | Future-use concepts | No | No | No | Yes |
| [44] | Patients | T1DM; unexpected glucose situations | Human-centered ML decision support | Longitudinal co-design/prototypes | No | Yes | Yes | No |
| [45] | Patients | T1DM and T2DM; self-management | AI-integrated wearables | Questionnaire-based prior or anticipated use | No | Yes | Yes | Yes |
| [46] | Patients, HCPs, and administrators | Diabetes care across the patient journey | Diagnostics, CGM, wearables, and chatbots | Settings with AI adoption | Yes | Yes | Yes | Yes |

**Abbreviations:** AI: artificial intelligence; T2DM: type 2 diabetes mellitus; EHR: electronic health record; HCP: healthcare professional; T1DM: type 1 diabetes mellitus; ML: machine learning; CGM: continuous glucose monitoring. “Yes” indicates that the study contributed data to at least one subtheme within the corresponding analytical theme. Detailed study
