# Supplementary material for: Stakeholder Perspectives on the Integration of AI in Diabetes Care: Systematic Review of Qualitative Studies
Source: J Med Internet Res. 2026 Sep 22;28:e105329. doi: 10.2196/105329 (PMC13596760; doi:10.2196/105329)
Supplement: Supplementary file 7 [file jmir-v28-e105329-s006.docx]

**Evidence profile table, GRADE CERQual (Confidence in the evidence from reviews of qualitative research)**

| **Summarized review finding** | **Methodological limitations** | **Coherence** | **Adequacy** | **Relevance** | **GRADE-CERQual assessment of confidence** | **References** |
| --- | --- | --- | --- | --- | --- | --- |
| **Theme 1: Enhancing clinical utility: prevention, management, and performance** | | | | | | |
| **Prevention, early diagnosis, and diagnostic precision** | | | | | | |
|  | **No/Very minor concerns** | **No/Very minor concerns** | **No/Very minor concerns** | **No/Very minor concerns** | **High confidence** |  |
| AI is perceived as a critical tool for shifting care from reactive treatment to preventive care, enhancing diagnostic precision, and acting as a safety net in high-paced environments. | High quality overall. Minor concerns in only one supporting study.[42] | Patterns of AI as a gatekeeper are compelling across all clinical roles. | Rich data provided by six independent sources. | Directly applicable to the diagnostic clinical encounter. |  | [34,35,39,40,42,46] |

| **Summarized review finding** | **Methodological limitations** | **Coherence** | **Adequacy** | **Relevance** | **GRADE-CERQual assessment of confidence** | **References** |
| --- | --- | --- | --- | --- | --- | --- |
| **Theme 1: Enhancing clinical utility: prevention, management, and performance** | | | | | | |
| **Holistic and individualized patient management** | | | | | | |
|  | **No/Very minor concerns** | **No/Very minor concerns** | **No/Very minor concerns** | **No/Very minor concerns** | **High confidence** |  |
| AI tools facilitate highly personalized and holistic patient management, encouraging the consideration of social determinants and mental health alongside glycemic metrics. | Supported by studies with Excellent[33,34,46] and Good[35,40] design fit. | Clear fit between the data and the conceptualization of individualized care. | Broad evidence base covering various patient profiles. | Directly addresses the aim of holistic diabetes care. |  | [33–35,40,46] |

| **Summarized review finding** | **Methodological limitations** | **Coherence** | **Adequacy** | **Relevance** | **GRADE-CERQual assessment of confidence** | **References** |
| --- | --- | --- | --- | --- | --- | --- |
| **Theme 1: Enhancing clinical utility: prevention, management, and performance** | | | | | | |
| **Time management and clinical safety** | | | | | | |
|  | **Minor concerns** | **No/Very minor concerns** | **No/Very minor concerns** | **No/Very minor concerns** | **High confidence** |  |
| AI functions as an efficient time management tool, reducing clinic congestion and bridging educational gaps by providing continuous support outside the clinical setting. | Minor concerns in three supporting studies.[37,41,42] | Consistent evidence regarding the reduction of clinic congestion. | High volume of illustrative data from seven studies. | Highly relevant to public healthcare system constraints. |  | [34,35,37,39–42] |

| **Summarized review finding** | **Methodological limitations** | **Coherence** | **Adequacy** | **Relevance** | **GRADE-CERQual assessment of confidence** | **References** |
| --- | --- | --- | --- | --- | --- | --- |
| **Theme 2: The role of technology in patient empowerment and self-management** | | | | | | |
| **Personalized education and awareness** | | | | | | |
|  | **No/Very minor concerns** | **No/Very minor concerns** | **No/Very minor concerns** | **No/Very minor concerns** | **High confidence** |  |
| AI tools serve as educational agents that adapt to cultural and personal realities, fostering greater patient awareness and ownership of their self-care journey. | High quality overall. Minor concerns in only one supporting study.[37] | Exceptional consistency on the role of AI as a trust filter for reliable information. | Supported by a large, heterogeneous body of evidence. | Directly captures the patient’s educational journey. |  | [33,34,36,37,39,46] |

| **Summarized review finding** | **Methodological limitations** | **Coherence** | **Adequacy** | **Relevance** | **GRADE-CERQual assessment of confidence** | **References** |
| --- | --- | --- | --- | --- | --- | --- |
| **Theme 2: The role of technology in patient empowerment and self-management** | | | | | | |
| **Decision-making support and real-life adaptation** | | | | | | |
|  | **Minor concerns** | **No/Very minor concerns** | **No/Very minor concerns** | **No/Very minor concerns** | **High confidence** |  |
| Access to real-time feedback and AI-driven reports supports decision-making and adaptation to atypical scenarios, such as illness, holidays, or intense physical activity. | Minor concerns in four supporting studies.[37,41,44,45] | High coherence across longitudinal [44] and experimental [36] data. | Very rich dataset exploring specific real-life challenges. | Highly relevant to daily Glc management tasks. |  | [33,35–37,39,41,44–46] |

| **Summarized review finding** | **Methodological limitations** | **Coherence** | **Adequacy** | **Relevance** | **GRADE-CERQual assessment of confidence** | **References** |
| --- | --- | --- | --- | --- | --- | --- |
| **Theme 2: The role of technology in patient empowerment and self-management** | | | | | | |
| **Motivation, discipline, and emotional well-being** | | | | | | |
|  | **No/Very concerns** | **Minor concerns** | **Moderate concerns** | **No/Very minor concerns** | **Moderate confidence** |  |
| AI acts as a motivational companion, helping patients overcome mental barriers and mitigate diagnostic-related stress through constant positive reinforcement. | High quality overall. Minor concerns in only one supporting study.[37] | Minor variations in how participants express their feelings | Data richness is lower; quotes are often superficial compared to technical themes. | Relevant to the psychological load of diabetes. |  | [33,36,37,39] |

| **Summarized review finding** | **Methodological limitations** | **Coherence** | **Adequacy** | **Relevance** | **GRADE-CERQual assessment of confidence** | **References** |
| --- | --- | --- | --- | --- | --- | --- |
| **Theme 2: The role of technology in patient empowerment and self-management** | | | | | | |
| **Support network: family, social, and clinical connection** | | | | | | |
|  | **No/Very minor concerns** | **No/Very minor concerns** | **No/Very minor concerns** | **No/Very minor concerns** | **High confidence** |  |
| AI resources act as communication bridges, fostering rapport between patients, family caregivers, and clinical teams while building a sense of shared community. | High quality overall. Minor concerns in only one supporting study.[37] | Consensus on the value of digital logs for family-based monitoring. | Sufficient evidence from four studies with deep insights. | Relevant to social support implementation strategies. |  | [33,36,37,46] |

| **Summarized review finding** | **Methodological limitations** | **Coherence** | **Adequacy** | **Relevance** | **GRADE-CERQual assessment of confidence** | **References** |
| --- | --- | --- | --- | --- | --- | --- |
| **Theme 3: Barriers to AI adoption in diabetes care** | | | | | | |
| **The trust deficit: accuracy, bias, and data security** | | | | | | |
|  | **Minor concerns** | **No/Very minor concerns** | **No/Very minor concerns** | **No/Very minor concerns** | **High confidence** |  |
| Trust remains the fundamental precondition for adoption, with primary barriers stemming from concerns about algorithmic accuracy, bias, and data security/privacy. | Minor concerns in four supporting studies.[41,42,44,45] | Findings are overwhelmingly consistent across 11 primary studies. | The most richly supported finding in the entire review. | Central to any AI implementation policy. |  | [33–35,38–42,44–46] |

| **Summarized review finding** | **Methodological limitations** | **Coherence** | **Adequacy** | **Relevance** | **GRADE-CERQual assessment of confidence** | **References** |
| --- | --- | --- | --- | --- | --- | --- |
| **Theme 3: Barriers to AI adoption in diabetes care** | | | | | | |
| **The irreplaceable human element and professional autonomy** | | | | | | |
|  | **Minor concerns** | **No/Very minor concerns** | **No/Very minor concerns** | **No/Very minor concerns** | **High confidence** |  |
| Apprehension exists that AI could erode the humanistic core of medicine. Specialists emphasize the importance of professional autonomy and human accountability. | Minor concerns in three supporting studies.[37,41,42] | Clear consensus on the irreplaceable nature of human touch. | Robust evidence representing multiple HCP types. | Directly addresses medical ethics and autonomy. |  | [34,35,37,39–42,46] |

| **Summarized review finding** | **Methodological limitations** | **Coherence** | **Adequacy** | **Relevance** | **GRADE-CERQual assessment of confidence** | **References** |
| --- | --- | --- | --- | --- | --- | --- |
| **Theme 3: Barriers to AI adoption in diabetes care** | | | | | | |
| **Increased systemic and caregiver burden** | | | | | | |
|  | **No/Very minor concerns** | **No/Very minor concerns** | **No/Very minor concerns** | **No/Very minor concerns** | **High confidence** |  |
| Poorly integrated AI systems may increase systemic burden through alert fatigue, data logging fatigue, and practical barriers related to digital literacy among elderly individuals. | High quality overall. Minor concerns in only one supporting study.[37] | Coherent findings across clinical (HCP) and home (Patient) settings. | Deep insight into the practical friction of technology. | Directly applicable to human factors and usability. |  | [33,34,37,39] |

| **Summarized review finding** | **Methodological limitations** | **Coherence** | **Adequacy** | **Relevance** | **GRADE-CERQual assessment of confidence** | **References** |
| --- | --- | --- | --- | --- | --- | --- |
| **Theme 4: Technical and educational requirements for successful implementation** | | | | | | |
| **Transparency and regulatory compliance** | | | | | | |
|  | **No/Very minor concerns** | **No/Very minor concerns** | **Moderate concerns** | **No/Very minor concerns** | **Moderate confidence** |  |
| Absolute transparency and regulatory compliance are essential to mitigate the "Black Box" phenomenon and ensure patient rights and liability protection. | Studies demonstrate high methodological quality and fit. | Strong coherence regarding the need for ethical/legal frameworks. | Supported by only two studies, limiting the overall richness of this subtheme. | Essential for institutional procurement and safety. |  | [34,35] |

| **Summarized review finding** | **Methodological limitations** | **Coherence** | **Adequacy** | **Relevance** | **GRADE-CERQual assessment of confidence** | **References** |
| --- | --- | --- | --- | --- | --- | --- |
| **Theme 4: Technical and educational requirements for successful implementation** | | | | | | |
| **Usability and technical preferences** | | | | | | |
|  | **Minor concerns** | **No/Very minor concerns** | **No/Very minor concerns** | **No/Very minor concerns** | **High confidence** |  |
| AI applications must be highly intuitive, visually engaging, and seamlessly integrated with wearable devices to automate data entry and prevent user fatigue. | Minor concerns in four supporting studies.[37,41,42,45] | Exceptional consistency across 12 studies regarding visual elements. | Extensive dataset with concrete technical feature requests. | Directly informs product design and engineering. |  | [33–35,37–43,45,46] |

| **Summarized review finding** | **Methodological limitations** | **Coherence** | **Adequacy** | **Relevance** | **GRADE-CERQual assessment of confidence** | **References** |
| --- | --- | --- | --- | --- | --- | --- |
| **Theme 4: Technical and educational requirements for successful implementation** | | | | | | |
| **Segmented education and multichannel support** | | | | | | |
|  | **No/Very minor concerns** | **No/Very minor concerns** | **No/Very minor concerns** | **No/Very minor concerns** | **High confidence** |  |
| Users advocate for a segmented learning approach and multifaceted, multichannel support systems, ranging from hands-on training to 24/7 hotlines. | High quality overall. Minor concerns in only one supporting study.[37] | High coherence across HCPs and patients on training needs. | Rich dataset exploring various support modalities (videos, hotlines). | Relevant to deployment and onboarding strategies. |  | [33,34,37–39] |

**Abbreviations:** Glc: glucose; HCP: healthcare professional; CAMELOT: Cochrane qualitative methodological limitations tool.
