# Supplementary material for: Stakeholder Perspectives on the Integration of AI in Diabetes Care: Systematic Review of Qualitative Studies
Source: J Med Internet Res. 2026 Sep 22;28:e105329. doi: 10.2196/105329 (PMC13596760; doi:10.2196/105329)
Supplement: Supplementary file 8 [file jmir-v28-e105329-s007.docx]

**. Quotations by themes and subthemes.**

**Theme 1: Enhancing clinical utility: prevention, management, and performance**

**Subtheme 1.1: Prevention, early diagnosis, and diagnostic precision**

No, we don’t already have a system. So I think there is value in adding a tool that would help improve care. [34]

...a lot of it [clinician decisions] is...individual clinician suspicion...a lot of it is going to be based on how well each clinician knows their patients. [34]

The primary argument for this tool...is that it’s easier to prevent something than it is to cure it. [34]

...the heart of what we do in primary care is to try to help patients with chronic conditions avoid long term complications of those conditions...if [AI believes] this person might be at greater risk, I might see [that patient] more often. I might spend more time with them. I might ask different questions because I would be trying to prevent [the complication]. [34]

AI aids in the early detection of diabetes, thyroid, endocrine system, obesity, and neoplasms-related issues, enabling timely intervention. [35]

AI triages and ranks radiology cases by urgency, enabling radiologists to focus on critical results first. [35]

AI identifies subtle anomalies in diagnostic images, facilitating early anomaly detection. [35]

Automated image analysis saves radiologists' time and expedites the screening process. [35]

AI leverages existing data to forecast disease trajectories and outcomes. This facilitates efficient prognosis and treatment planning. [35]

AI analyzes extensive medical literature to identify new trends and potential treatments for metabolic and endocrine diseases. [35]

AI analyzes molecular data and predicts potential therapeutic candidates, supporting drug discovery and development processes. [35]

For junior people, AI can help make better diagnoses. For diabetes, AI can remind you to do necessary checks like eye and foot examinations. [39]

[The most helpful aspect of conversing with the AI was the] ability to interact with it to gain further insights and clarification on the data provided. Being able to ask follow-up questions on things like how certain factors influence risk or why certain factors were included helped me better understand how the AI was processing the data. [42]

Provides a quick and concise overview of factors contributing to the individual’s risk of diabetes. [42]

Allows the practitioner to clearly see what areas are concerning and what areas are acceptable. [42]

...it...makes you think twice...it...makes you pay attention a little bit closer, and makes [you] ask, okay, why are they at risk? What are the things that I can do to reduce the risk? [34]

We’ve really focused on making our diabetes care more patient-centered. By creating a comfortable and inclusive atmosphere, we make sure our patients feel supported every step of the way. We’ve done things like hiring staff from diverse backgrounds and giving patients online access to their medical records. This has improved their overall experience and helped them manage their diabetes better. Simplifying appointment scheduling and clearly communicating our policies have also made our patients feel more empowered. [46]

We prioritize giving our patients access to various medical specialists because we believe a team approach is key to managing diabetes effectively. Patients can easily schedule appointments and access their records online, which has made their care experience smoother. By creating an inclusive environment andoffering excellent medical support, we’ve seen an improvement in patient satisfaction. Patients feel more confident and informed about managing their diabetes, and this has positively impacted their overall well-being. [46]

AI has really improved our diagnostic accuracy and efficiency. We can now analyze patient records, test results, and imaging data much faster and more accurately. Our continuous glucose monitoring systems provide real-time data, which helps patients maintain better control of their blood sugar levels. [46]

I like the idea of the tool as a gatekeeper for patient safety. Making sure doctors don't forget things, reminding us to check and act on abnormal results. I think that is useful for busy clinics. [40]

AI-driven alert systems prevent serious hypoglycemic episodes and ensure patient safety. Patients feel empowered with access to informational materials and data-driven insights. [46]

I find that for patients who are diabetic, it is the frequency of touches at every opportunity to control their diabetes that makes the biggest difference. And so if a patient has come in for a cold, or even anything else, other than diabetes, there’s an opportunity to intervene. For those patients who are poorly controlled, it’s usually because they’re engaging with a system very infrequently. And so from that perspective, getting them reengaged in the system to become familiar with a system becomes the most valuable tool. [34]

...as a clinician, it’s part of my responsibility to have some awareness of the...health...of...my small population...And so this would help to do some of that. [34]

And also, it’s part of our billing, and HEDIS measures anyway, we’re supposed to have A1cs that are below eight, and so I feel like this is designed to meet that standard. [34]

One of the biggest benefits of AI is its ability to support clinical decision-making and medication development. It also helps with population health management and patient engagement, providing valuable insights that improve overall care quality. [46]

**Subtheme 1.2: Holistic and individualized patient management**

Now that [AI] has brought it up...I would explore things...that cause high A1c’s like social determinants, depression, medical intensification... [34]

I think it would be very useful, because it really takes a kind of a holistic approach of looking at the entire patient, and not just, I’m not just looking at like their blood sugar. [34]

I would provide education about the connection between depression and diabetes, and how they can very much go hand in hand, and how a diabetes diagnosis can either lead to a depression diagnosis or exacerbate depression that’s already there. [34]

AI examines patient-specific data and provides precise therapy recommendations based on individual traits and disease profiles. [35]

AI suggests personalized interventions based on patients' medical history, genetics, lifestyle, and treatment response. [35]

AI recommends optimal drugs and treatments for various metabolic and endocrine disorders. [35]

AI analyzes extensive datasets and medical literature, enabling treatment decisions based on the best available evidence. [35]

AI-powered chatbots offer individualized education on treating metabolic and endocrine issues, including diet and lifestyle recommendations. [35]

I think the most valuable part for me is the lipid control feature. Sometimes when engrossed in discussing patients' diabetes treatment plans, which is anyway their primary reasons for seeking consultation, I may overlook the assessment of their LDL-c levels. With the APA tool, a quick glance provides a clear indication of whether they are on target or not. There isn't much extra clinical information that is required by the tool, so I am able to rely on the medication recommendations to appropriately adjust the medication for hyperlipidemia. [40]

AI helps us personalize treatment plans for each patient. We can predict complications and provide remote monitoring and telemedicine services. This has made managing diabetes much more efficient and effective. [46]

AI has allowed us to tailor treatment regimens using individual patient data, which improves glucose control and overall health. [46]

We expect advancements in personalized treatment, predictive analytics, and telehealth solutions. [46]

AI assists medical professionals in managing metabolic and endocrine diseases in line with accepted clinical standards. [35]

AI provides patients with support and reminders to help them adhere to treatment regimens and prescribed medications. [35]

AI evaluates treatment responses to predict the effectiveness of specific interventions for individual patients. [35]

If the patient really cannot decide for herself and a caregiver comes, this is a very good thing. The caregiver can look out for the food consumed and give the medicine when there is a missing dose. For a caregiver with a patient who is unable to reach out for her own medicine or anything like that, this is a good app I must say. [33]

…I think some patients don’t use the phone regularly, especially for elderly patients, which will be a bit more difficult to manage condition through the app. It's good that there's an extension to the family members who can help with all these things. I think it's a good idea. [33]

I think definitely…in [an] underserved population, it might be more beneficial, especially since they have less access to care. [34]

The whole predicting, based on community or...based on where the person lives...struck me a little odd...it feels almost like...an overgeneralization...[because] you come from this community, you are at risk...Are we stereotyping?...Are we making assumptions...because someone comes from...poverty, or...a certain marginalized population? [34]

I would probably focus on the people I know who already have A1c’s more than 9% and start working on that population first. [34]

...a lot of it is going to be based on how well each clinician knows their patients, and how well and how comfortable the patient feels and speaking up on their own behalf for concerns that might have arisen. [34]

**Subtheme 1.3: Time management and clinical afety**

It’s the way forward because it can reach many patients simultaneously. They can use it at their convenience, with different modules. It’s a very good approach. [39]

I think generally AI is an advanced technology and good for people. It helps us in many works. And I think in medical also very useful, can save time energy. [41]

I personally belief AI technology can be a good alternative to treat patients remotely. We old people very hard to come for appointment every month because no parking, crowded. Hence, if we can use AI to monitor patients, it will be easier. [41]

We don’t see patients daily or even weekly. At most, it’s monthly or every three months. That’s not enough to educate them sufficiently. A mobile app would give them more time to learn daily. That would be more effective. [39]

Education is crucial in diabetes management. At the clinic, we have limited time for education. Patients often don’t retain information from consultations, so it’s good to have something outside the clinics. [39]

Nowadays, hospital have lot of congestion issues, especially HTAR. Too many patients. So maybe with this AI, we can reduce this congestion issues. Like the doctor monitor patient through apps and patients no need to go to hospitals for normal check-ups, like in overseas. [41]

I tell you if hospitals use AI in treating patients, it will be big relief as there will be not much patient waiting issues in the hospital. Easier and faster treatment method. [41]

These recommendations would be more valuable in a primary healthcare setting, where doctors may not have extensive knowledge of clinical practices related to novel glucose-lowering medications and insulin titration, especially in complex cases. I think implementing the AP tool in such settings would greatly help doctors in improving patient engagement and care. [40]

The AI was able to give a summary of the data in a simple to read and understand format whereas I would have to read through the data and come to a conclusion myself. The AI doing it for me helped in this process. [42]

[Knowing which patients are at high risk is] kind of helpful...[it tells you] where to put your resources. [34]

AI seamlessly communicates data with existing hospital systems, optimizing workflow efficiency. [35]

AI reduces unnecessary procedures and testing, resulting in cost savings for the healthcare system. [35]

AI identifies high-risk patients and enhances treatment plans, enabling more effective resource allocation in the healthcare industry. [35]

Let’s just say that most of the time, doctors just throw you a chunk of information and then you're supposed to go home and digest it. Then, digestion or indigestion is another issue…so I am open to it [MI]. It’s something that will benefit me. [37]

Well, for my case, I would prefer an app [based MI] because I can do this like, anywhere. During my lunchtime, I can do it while I am at my work. [37]

**Theme 2: The role of technology in patient empowerment and self-management**

**Subtheme 2.1: Personalized education and awareness**

I love the feature where you set the goals…at least at the end of the day, we can discuss what we ate for the day. Actually, sitting down and talking to family members takes time and sometimes they are quite busy, but by having this app, we can look through and make time to discuss.[33]

With this app, I can monitor a bit more because without it, I don’t really know about the diet and physical activity record. The medication, most of the time, I don’t actively monitor. It's just that I know that she's regular about it. If she misses, sometimes she’ll tell me but if she never says, then I don’t know. With the app, it can tell me more, I can play a more active role.[33]

I can see what she's having for lunch then I can remind her maybe at dinner time not to eat too heavy…So this app will be good for the family members to personally look out for the patients… For many diabetic patients, food intake is very important so if it can give a very accurate analysis…then it will be very useful. [33]

[Medication logging] is good because sometimes when I go to hospital, the doctor asks me the medication my wife takes. Instead of verbally telling the medication, I can show the app…easy for the doctor to understand. [33]

...awareness is probably some of the best medicine you can give. And my philosophy is empowering a patient to give them the education, so they can make better decisions moving forward...I’m trying to empower this patient to take control of their own care. [34]

I became aware that medication management is an important aspect of DM management because it can help to regulate blood sugar levels and reduce the risk of short-term and long-term complications. Also, by adopting lifestyle changes such as exercise, balanced diet, we can reduce the medication intake after consultation with the physician. However, the decision can be taken after monitoring the glucose levels for a period of time. The application also suggested real-time glucose monitoring patch powered by AI technologies, which can monitor the glucose levels 24×7 and change diet and medication accordingly. [36]

When I asked a sample vegetarian diet, the application gave me a meal plan for breakfast, two-time snacks, and dinner. It recommended oatmeal along with nuts, berries, and non-dairy milk for breakfast; raw boiled vegetables for afternoon snacks; and, for lunch, items such as grilled vegetable sandwich on whole grain bread with avocado spread; Greek yogurt for evening snacks; and tofu stir-fry with brown rice and steamed broccoli for dinner. The meal plan was very much customized according to my requirements. [36]

I have cardiac problems and also DM; as a result, I may have to be careful in doing physical exercises. When I asked the application, it initially suggested to consult physician, but also suggested some customized routine. It suggested low-intensity exercises such as walking or cycling and gradually increase the intensity and duration over time. In addition, it suggested low-impact exercises that are gentle on the joints and minimize the risk of injury, such as swimming, yoga, or tai chi. [36]

The most interesting aspect is routine. It suggested five to 10 minutes for warm-up exercises such as brisk walking before indulging in any physical exercises and later cardiovascular exercises, such as yoga, tai chi, followed by cool-down exercise such as brisk walking. [36]

When I asked for an exercise plan, ChatGPT suggested brisk walking after Iftar during Ramadan, instead of during fasting hours, which I found practical and manageable. [36]

I was given different recommendations and diet routines based on my likes and dislikes for food types. I informed that I like spicy foods. Based on my requirements, the application offered me different routines that included Mediterranean, DASH, and low-carbohydrate diets. [36]

Diet wise, I would prefer more app-based MI because it can be individualized. I have been advised not to eat this and that [from healthcare professionals]. I get frustrated because it's like someone keeps telling me to avoid certain food, which then becomes my own problem…I’d like to get advice through app on what I can eat or why I can't eat. [37]

I was not aware about the importance of exercise for managing DM. However, after interacting with the application, I came to know that exercises can improve insulin sensitivity. Moreover, the application also presented different types of simple exercises such as stretching, cycling, dancing, and jogging. Brisk walking of 2000 to 4000 steps was suggested as it is suitable exercise for all those without ortho problems in the legs. [36]

I didn't know much about how different foods affect my blood sugar. ChatGPT explained the importance of the Mediterranean diet and suggested specific meals like grilled fish with olive oil and whole grains. This was very helpful because it aligned with my cultural food preferences. [36]

The application helped me in learning about the importance of monitoring blood glucose levels. I got to know from the application that when blood sugar levels are too high, it can cause damage to organs and tissues over time, leading to long-term complications such as nerve damage, kidney damage, and eye damage. When blood sugar levels are too low, it can cause immediate symptoms such as dizziness, confusion, and sweating and can be potentially life-threatening. [36]

The application provided links to various educational resources from American Diabetes Association (ADA), Diabetes Self-Management Magazine, National Diabetes Education Program, which were helpful in recognizing the reliable educational resources. This helped me to stay away from the resources that provide mis-/disinformation about DM and its self-management process. [36]

ChatGPT recommended the American Diabetes Association's website and research articles. Now I know where to find reliable information instead of random social media posts. [36]

It is a very good powerful supporting tool in diabetes education, especially for patients who can’t access a diabetes educator or DMTAC pharmacist. For hospitals without these services, dedicated patients can find support through these mobile apps. It is very convenient. [39]

Remote monitoring and self-management apps have enhanced how patients interact with their health data and manage their disease. [46]

Data-driven insights from AI help patients advocate for their needs and take early action on their health, leading to better outcome. [46]

AI can analyze patient data to create personalized treatment plans and provide real-time feedback and monitoring, which greatly enhances patient care. [46]

Many patients find AI solutions convenient as they allow quick adjustments to treatment strategies through real-time feedback and monitoring. [46]

Patients appreciate access to data-driven insights, personalized treatment plans, and improved interactions with healthcare professionals. [46]

Closed-loop systems and behavioural insights will become more precise, enhancing overall patient care. [46]

AI has allowed us to provide personalized instructions and real-time feedback, which has significantly improved patient engagement. [46]

Remote observation and telemedicine have become more effective with AI, improving patient education and engagement. [46]

**Subtheme 2.2: Decision-making support and real-life adaptation**

I logged my blood sugar levels daily and asked ChatGPT if I needed to change anything. It suggested increasing fiber intake and monitoring for another few days before seeing my doctor. This helped me make informed decisions. [36]

The categorization of different types of physical activities and exercises really helped me to better understand the need for structured exercise plans, and accordingly, I was able to make better decisions on selecting the type of exercise for a particular day. [36]

It will make patients take ownership of their own self-management, likely making it more effective. [39]

AI in mobile apps are powerful for people with diabetes as they can take control of their own management. AI can help them make decisions about activity and diet by providing lots of information. [39]

I did not know about CGM patches before using ChatGPT. It explained how they track glucose levels in real time. Now I use one, and it has made a huge difference in how I manage my DM. [36]

I started using a continuous glucose monitoring (CGM) device, and I would input my daily readings into ChatGPT to ask if my glucose levels were stable. When I noticed that my readings were consistently high in the mornings, ChatGPT explained the possibility of the dawn phenomenon and suggested adjusting my evening meal and consulting my doctor. This helped me understand my patterns better and make small changes to improve my blood sugar control. [36]

I was not aware of the continuous glucose monitoring process. The application suggested me continuous glucose monitoring (CGM) device, which is inserted under the skin, usually on the abdomen or upper arm. The sensor monitors the glucose levels continuously and transmits the data to the receiver's device which can be accessed from the application. I contacted my physician about it and got a CGM patch for me, and now I can check my glucose levels in real time. Thanks to ChatGPT for providing such important information. [36]

The mobile app can assist patients in changing behaviour because we can monitor them. Patients have all the information, so they become aware and change accordingly. AI can track patient data, efficiently changing their behaviour. [39]

Real-time monitoring and feedback provide convenience, allowing quick adjustments to diet, exercise, and insulin doses. [46]

The AI app gives me clear feedback on my glucose levels, making me feel more informed. [46]

AI reminders help me adjust my diet and insulin doses quickly. [46]

I feel safer with AI monitoring my sugar levels daily. [46]

It would be helpful to remind me about my insulin doses. [45]

Wearable devices can help me remember doses when I’m busy. [45]

If the device could alert me to refill my medication, that would be helpful. [45]

Looking at the specific scenarios, say, illness, you’d be at work, you’d start to feel ill but you’re not really sure whether you are getting ill, you won’t know until the next morning usually, and then you get to the next morning and you wake up and then yes, you’re definitely ill now. Then you would tell the app you’re ill, and then you can work back in retrospect, so you can tell it when you started feeling ill and then it would segment that data separately to your regular data, so then it’s not collecting that data as a whole, it’s collecting it like, ‘This is your illness data’, ‘This is your holiday data’, so it won’t work the same as your basic data, so it’s not drawing information based on when you’re ill, and the algorithm learning from that, you’re giving it different contexts so it deals with it differently. [44]

So, for this [scenario] you get up in the morning, eat your breakfast. Chuck it in the app and take insulin based on that, and you’ve already planned to go to the gym. So, then you’ve cycled to the gym. Then as you’re cycling you get an alarm – you get hypo and then you have to re-adjust [because the app gave you a wrong insulin dose suggestion]. [44]

It could be seen as not working but working because if this is happening when the app is kind of getting to know you, then it could be taking on this information and if you go through the same scenario again at a later date, then it’s kind of learnt and can maybe make suggestions about how many carbs you might want to think about taking before going to the gym to avoid having a hypo. Part of the learning process. [44]

A lot of the stuff you eat [during Christmas] tends to be quite sugary. You’re not doing much exercise, you’re usually sat down for a large amount of the holidays. So, the way you’re talking about routine, Christmas is a bit of a blip in a routine really. For me anyway, it’s still trying to look at labels and just dose accordingly and sometimes you get it wrong because there’s other things at play like not being active is going to have an effect as well. [44]

Report card is good because we're busy, we like to see things at a glance, like a summary. Like for October, this is your average readings, and you can kind of improve, then if you've not been doing too good in October, then you tell yourself, okay, November, I'm going to walk more, I'm going to have to bring my blood pressure down a bit more, things like that. [33]

The report card, I really like that too…it's easy for me to know how well she is doing. Because right now, if you ask me to recall how she's doing, it is difficult to tell you that quantitatively. But here when I look at the card, I can see for myself that over the month, what the changes are that she has made or even if she is keeping it constant, that's also fine. It's better than going downwards. [33]

…the systolic and diastolic…give us a brief on what the number means and an acceptable range to be in… because we wouldn’t be able to tell whether 130 is a good range or out of the range. [33]

ChatGPT asked me a series of questions about my lifestyle and gave me a risk score for developing complications. It made me realize I needed to be more proactive about my health. [36]

I asked the application if it can assess the risk of getting DM. It stated that it can, but it wanted me to answer few questions like age, eating habits, lifestyle, etc. I answered them, and it accurately identified that there is high risk for me to develop DM. [36]

**Subtheme 2.3: Motivation, discipline, and emotional well-being**

Frankly, speaking, I am really looking forward to all these [features] because sometimes, discipline is very important. I got no discipline. So, it's good to have this [family module].[33]

I think it's a very good idea to connect the two of us, so it's not just in silo, I will know what she is doing, and I guess we can motivate each other to have a healthier lifestyle in terms of workout and stuff, to be more active.[33]

MI would be good to overcome mental barriers. MI can serve as a check-in mechanism to remind me of my progress and how to improve [my behavior]. So even when I am tired, I will still make an effort to exercise. [37]

I would like to have somebody that I can talk to because he or she will understand what I could eat or what I could do, that will help lower my cholesterol or improve diabetes. [37]

AI will be a motivational tool to change behaviour. Constant motivation is key for behavioural change, and AI can provide that continuous motivational input. [39]

The system should share trends in behaviours, like tracking mental health status and correlating it with eating habits and blood glucose. This helps patients think about lifestyle changes and come up with solutions to reinforce behavioural change. [39]

I was very stressed that I was diagnosed with DM at a very young age. Suddenly, I had to modify my diet, daily routine, and many activities, which made me to stand out among my friends. As a result, I had to undergo lot of stress. But ever since I started using ChatGPT, I realized the various ways it can help me to cope up with the emotional stress. It provides ideas of relaxation techniques such as yoga, meditation. It suggests me physical activities to indulge in which can help both my condition and stress. [36]

Managing DM is stressful, but ChatGPT taught me deep breathing exercises and stress relief techniques. It even cracked a joke when I said I was feeling down! [36]

The most difficult thing is that patients need self-motivation or self-discipline to follow the mobile app’s instructions. [39]

To motivate patients who feel life is meaningless if they can’t eat freely, the app can show pictures of complications like amputations. [39]

Building rapport with patients is crucial so they trust what we share. It’s important before we educate them, to help them understand and change behaviour. [39]

**Subtheme 2.4: Support network: family, social, and clinical connection**

…because I’m not with my wife during lunch time so with this, at least I know whether she has taken her medicine for lunch or not. So, I can see and then if she hasn’t, I can remind her. [33]

I think for me, it [module] is more for myself to remind myself and I already manage quite well. I don’t have any issues for people to remind me. [33]

When I asked the application if it can remind me about medicine intake, it stated that it cannot, as it is an AI language model. However, it said that there are AI-based eHealth applications that uses its models which can provide recommendations and reminders by monitoring the blood glucose levels. [36]

Thanks to the study and the application. The application was very engaging, and it helped me in learning many new things about DM self-management. Earlier, I used to just follow what doctor says, and I don't know what to ask, as I was not aware about my disease. But now, I can ask many things like my exercise regime, diet plans, and many things that help me in better managing the blood glucose levels. [36]

Before my last check-up, I asked ChatGPT what I should discuss with my doctor. It suggested asking about insulin dose adjustments and diet changes. I felt more confident in my appointment. [36]

When I asked how can it help me in communicating with my healthcare provider, it stated that it can help them schedule appointments, provide updates on their condition, and ask questions about their treatment plan. [36]

Good communication and patient education are crucial for us. We’ve invested in technology to improve how patients and healthcare providers communicate. We have educators who offer personalized support and make sure patients get the resources they need. We also regularly ask for patient feedback to improve our services. This patient-centered approach has built trust and a sense of partnership between our patients and our healthcare team, leading to better health outcomes and overall satisfaction. [46]

AI systems have improved communication with healthcare providers and provided patients with valuable insights for better disease management. [46]

I was informed by the application that it can be integrated with online platforms in order to facilitate DM patients to connect with one another and share their experiences. [36]

I never knew there were Arabic-speaking DM support groups. ChatGPT recommended a few, and now I have a community to share experiences with. [36]

I was asking the application for DM self-management groups. It provided many groups such as 'DM daily,' 'Beyond Type 1,' 'T1D Exchange.' It helped me to come in contact with many other DM patients by sharing original sources, links, and also shared many informational messages. [36]

We can do it together now, and we can talk to each other and share anything under the roof. So, the time we exercise together, we also can build our rapport and our relationship better, I think that really helps us. We can share our agreements and disagreements. [33]

If you’re alone, sometimes a bit lazy and tend to slack off. [Goal setting] will be quite encouraging but it also depends on family. Sometimes kids are busy studying and my wife is busy. Then I cannot force them. [33]

If we set ourselves, it might not be enough. If we set too difficult, then that's fine but if we set too low, then not enough. The AI can give a reference, then based on the reference, we adjust, that might be good. [33]

If it's just long term, the focus might be lost. I prefer short-term goals because as of right now, like checking the glucose, the patient can see little successes. But long-term goal is good to get the overall picture, the holistic picture. But the short-term goals can help to motivate them to have a better, healthy lifestyle. [33]

Because sometimes face-to-face you want to say something, but you cannot articulate. That's something I am worried about, like offending someone. So, this [app] is better. If I am not happy with what I will say, I don’t have to mention immediately in the app. [37]

**Theme 3: Barriers to AI adoption in diabetes care**

**Subtheme 3.1: The trust deficit: accuracy, bias, and data security**

I don’t do anything at work, I literally sit down at a computer for eight hours, very little movement and so I have my lunch, I have a salad for lunch, no carbs in that anyway, so generally don’t do an injection with that, so work is pretty simple to control, it’s just every other bit of living is awkward. [44]

I probably wouldn’t use [a decision support app] in everyday life, because I’ve been diabetic for 35 years so there’s not much I would need to be told how to do it. But things like going on holiday, different time zones, exercises, that would be useful. I’d find it really useful. [44]

Yes, and I think it does depend on where you are, how long you’ve had diabetes. For people who are only recently diagnosed, I think this can be very, very helpful, but as you say, I’ve been insulin-dependent for 30 years, and I know what I’m doing most of the time. [44]

Would it make care worse? Yeah, potentially...So if you’re prompted to prescribe medications...for people who are not yet at a certain level of risk, the [benefit to harm] ratio becomes smaller. [34]

If it’s things that are [inaccurate and] manually entered into the EHR system that are driving this..., it certainly could create false alerts and waste time or...miss people who actually are at risk because...things weren’t...entered correctly, or left blank. [34]

AI algorithms might fail to consider the larger clinical context, leading to inaccurate results or suggestions. [35]

I worry about inaccurate information. This can happen if inaccurate information is fed to the AI, like patients not giving truthful information. [39]

I’m worried about the accuracy of the data the device collects. [45]

Sometimes, I see discrepancies in readings. [45]

I am concerned that the device might malfunction during a critical time. [45]

I’ve had devices stop working mid-day.

...the primary concern stems from excess information being available...But if there’s already a lot of data points, and they’re not...actionable, it can be overwhelming or just ignored. [34]

We are asked on a monthly basis to review our patients who are not at a goal hemoglobin A1c level. Our...focus in the last six months has been...around Latino patients...So I find...this particular...information to be less valuable because we’re kind of doing it on a monthly basis already. [34]

You have to prove to me first that identifying and managing folks like this can actually help. [34]

It’s only useful if I trust the information. [34]

We can use AI but cannot completely because we scared sometime the result or diagnosis from AI will be wrong or some errors. Especially in medical diagnosis, worrying about that. [41]

People like me don’t know much about AI will have problem if we use AI in diabetes care. [41]

I don’t know how government can include AI in health care system. It must be troublesome. Must make sure no errors occur. [41]

Don’t want to entrust someone’s health fully with a computer. [42]

It [scientific evidence] slightly increased trust, but just because a paper is referenced does not mean that it is scientifically robust or clinically significant, I would want to review the source myself to see if it was trustworthy. [42]

I guess you’ve gotta build a bit of trust and confidence in it all the time. [44]

Some of that’s to do with the data you put in, isn’t it? So, if you know you’ve skipped things you might not trust it whereas if you know that you’ve put in – you can trust your data but then there’s the data just to test so, I guess it’s building trust in that data. [44]

Keep doing it until you trust it. [44]

And I suppose you’d have a gut feeling as well. If what it’s telling you seems completely wrong you probably wouldn’t go with it. Whereas if it seems... if you think it’s... well, you’d probably be more likely to. [44]

At first it was hard for me to kind of trust it a little bit, because I was so used to doing the finger stick. And I just wasn't sure what the technology, what was going to happen? [38]

One concern is that some patients don’t fully understand how AI algorithms work, which affects their trust in the technology. We need to be more transparent about how AI makes decisions. [46]

Workflow disruptions and data accuracy concerns were significant. Clear communication across departments was crucial to overcoming these issues. [46]

Some patients don’t fully understand how AI algorithms work, which affects their trust. [46]

But it’s like if you’re eating a snack but you don’t tell it you’re eatinga snack and then it recognises an anomaly then could it just pop up and say…[44]

Because then it could say like, as you said, ‘did you have a snack?’ and you said ‘no, I didn’t have a snack’ it could be like you’re getting poorly or you’re really stressed or you don’t know. [44]

Yeah, so the quality of the data you put in. But again, I think at the end of the day almost you could almost sort of say ‘oh, I was a bit rubbish today, I didn’t tell you much today’ or ‘I’ve been really good, I’ve told you everything today’. It’s almost like... so you could almost say ‘oh, yeah, this day isn’t very good to use as an example’ or ‘this day is a pretty good day’, so you can almost just like say ‘just forget about some data’ maybe. [44]

If there's a third-party device or app, I'm more interested in it. If there are ways you can change it or look at the information that it's going through or if it can be calibrated, if it's put out by the FDA or if it's something put out by a specific company or reputable company, I'd want to look at the research data. I'd want to see how many poor outcomes there were. I'd want to see what the reliability is and the accuracy of it, but the user information too. [38]

Because not everybody's diabetes is exactly the same. But it would be nice to see this person is getting really different results from me that are so different? They're not even proportional. What's going on with mine? Is there something broken? [38]

I trust my doctors enough [to select device for me]. [38]

I trusted everything because of my doctor. It took me the first day [to trust the device] I just trusted. [38]

I mean before a [device goes to] market or a product even hits the market. Typically, they do a whole bunch of testing. But it would be nice to know. You know this has been proven to work. This is like you can trust this. [38]

I may be less likely to jump into new technology because I have the other technology I can compare it to. So, I'm using older information like we have our fingerstick glucometers that we can check and compare to our readings on a CGM or FGM. So, if there's a backup or something that you're familiar with to compare it to the new stuff, I think that makes it a lot easier to adapt. [38]

I think that would depend for me if is this a machine learning? Does it learn the individual? If so, then yes, absolutely the diversity of that sample and how that machine learning algorithm actually works across groups is important. But if it's a more basic calculation that is kind of an already existing goal standard, then maybe less so, because if that would've happened on the back end in the development of that standard that exists. [38]

I want to make sure that that algorithm has been tested well in randomized control trials, to make sure that we're not getting. erroneous doses and stuff, especially when the AI technology taking over your dosing that's where the [glucose] level goes up to like get 10 but for something like in pen where it's really just a calculator taking all the data and doing the math for you, I don't need that tested I just need to know the math works. [38]

[In order to trust this device, I want to know] If there are, people [like me with my type of diabetes who] have actually used this device and [results]. [38]

So, I prefer to have more personal reviews rather than the manufacturers like specifications. [38]

I always want to know if someone else has used it, they've used it and what their opinion is of it. [38]

I feel like there needs to be a database for the past experiences of people that have used the app and how it worked for them. [38]

Did you just develop the app and never tested it on anybody and you're going to roll it out now and let people buy it and we don't know how accurate it is, or did you actually

go through a process whereby you tested the product and it is comparable to others that may be in the category. [38]

I [provider] don't know that patients pay so much attention to its [the devices] accuracy. [38]

I don't know that people using the devices necessarily think about that at all, and I think people who use the device care about them and how it is accurate or not accurate in them personally. [38]

But if there are limitations to the accuracy, or if there are known things that would interfere with the readings, or the recommendations for some reason, so if there are limitations to the application. Suggest sharing that [information during training]. [38]

.. having something on the label that says the American Diabetes Association is a partner or approves it or you can talk to someone. [38]

Then another thing that is important to me is if it's FDA approved, or not like if it's [the device] something that's vetted that way, then I trust it way more than I would trust something that doesn't carry that level of credibility and for me. [38]

I also think things such as American Diabetes Association. [38]

Like diabetes group sources, like VA or the CDC educator groups where I got my information. [38]

I rely on my research but I, ultimately me, it really is going to be on the medical professional. [38]

I [rely on my healthcare provider]. [38]

I just trusted everything because of my doctors. [38]

[my source of information is my] Provider, other people with diabetes. [38]

Like you guys are doing it just. I just trust you guys [pharmacists]. [38]

My number one [source of information] is my provider. [38]

I mean the manufacturer site if there's some great FAQs or whatever like that's the first place, I would go and look and then, just forums online of what other people have found. But yeah, usually I start with the manufacturer. [38]

When it [APA] was launched, a lot of us were not very sure how it was developed. I think part of the reason why we did not use it very much is also because we are not so familiar with how this system came about, what kind of information was used, and where the information came from. Is it also possible that critical information was not captured in the system? I can't trust totally, and [I am] not confident with what I'm seeing at the moment. [40]

That being said, if more information or transparent communication is given to us, I might be more inclined to use it in clinics. As I know the logic and rules behind these recommendations and where they are sourced from. [40]

I have a lot of confidence in the scientific ranges and I don’t know enough about the datasets the AI is using to know how accurate its assessment is. [42]

Just for my doctors. [38]

I want to get it [information] directly from my doctor. [38]

When considering different devices, the best way to share information is a diabetes website. [38]

Most of it has been googling and finding forums, and what people have said and talked about their experience. Obviously, that's going to be a different level of credibility than manufacturer or my doctor, but if other people have had a similar experience, I think it has some value. [38]

I would be concerned about [the] over identification [and] over diagnosis. [34]

Racial bias is...something that’s implicitly existent in normal data sets...this is something that just compounds...It’s like a small mistake that compounds into something bigger. [34]

If AI algorithms are trained on biased datasets, they may draw false conclusions and perpetuate healthcare inequities. [35]

We need to address biases in AI data to ensure fair conclusions. There’s also the challenge of accountability when AI systems make errors. Balancing AI and human healthcare providers is essential. [46]

If AI models are trained on specific datasets, they may not represent the full spectrum of patient populations. This can undermine the accuracy of the results for some groups. [35]

Disparities in healthcare infrastructure and socioeconomic status affect access to AI-based interventions. [46]

Integrating AI systems with our electronic health records was a major challenge. We had to redesign workflows and provide extensive training for our staff. [46]

Efforts include extending telehealth services, promoting digital health literacy, and designing culturally sensitive AI tools. [46]

AI systems may be vulnerable to data breaches, posing a risk to patient data and privacy. [35]

The application of AI in medical decision-making may raise liability and malpractice issues, especially if unfavorable outcomes occur. [35]

It's nice to know the [device safety] limitations that there are significant limitations. [38]

[safety need] something that would detect that the sensor was bad. [38]

would start erroring and say hey you can't trust your Dexcom. [38]

Data security is a major issue. Collecting and storing patient data raises concerns about privacy. [46]

I would want to be darn sure that this thing [device] would never be able to [unexpectedly drop my blood sugar] do that. [38]

Like a Max Bolus feature where it's like, “You can't give yourself more than this.” [38]

So, I know with the pumps that are the closed hybrid group systems, most of them uh, because they talk to the sensor, have in those safety mechanisms whereby if there's a problem with the sugar's dropping, it's simply suspends the insulin dose. There's an in-built algorithm for safety. Or if someone's not doing something right, the sugars are high for hours and hours it goes into a safety more. Almost all the pumps have that these days. So yeah, I mean, I don't know if this needs to be that sophisticated. [38]

Also, are they [patients with diabetes are] going to have the ability to override I think we always need the ability to override if things are low, just to stop. [38]

formal safety training might be helpful, but I just want to figure things out as I go. [38]

A training [about alerts] for sure, would be beneficial, and then, honestly, like a little check card [with all the alerts and their meaning], would be kind of nice if it was just like a little index card or something like that. [38]

If the Dexcom is not reading correctly, I would hope it would just say the pump formal safety training might be helpful, but I just want to figure things out as I go. [38]

A training [about alerts] for sure, would be beneficial, and then, honestly, like a little check card [with all the alerts and their meaning], would be kind of nice if it was just like a little index card or something like that. [38]

But I would want to know if I could do it [shut off the device] manually, if something went wrong, and I'd have to enter by hand if I could do that or not. [38]

Privacy is a concern. If patients feel unsafe using the apps or recording data due to security issues, we need to reinforce privacy measures. [39]

Long-term, we need to consider legal implications of using this data. We must address data security and patient privacy. [39]

I definitely think, having the capability to share [device information and readings] is good, because not everyone will want to share, but especially in the case of like a child and a parent, that that child Isn't, always going to know what to do, or especially depending on their age. [38]

Data security is a major issue. Collecting and storing patient data raises concerns about privacy and potential misuse. [46]

Resource allocation, privacy, and security were challenging. Ensuring regulatory compliance and managing patient expectations required careful planning and continuous maintenance of AI systems. [46]

No worries about data privacy. We have used to many technologies. [41]

Not much very about the privacy all because I believe the benefit is more actually. [41]

Nobody, no one [I don't have a problem with sharing my information]. [38]

Yeah, let the manufacturer have the information so they could make the technology better. The more they know the better. [38]

[I am ok sharing formation with the manufacturer as] long as it doesn't infringe on my safety and security. I mean, just information, that's fine. They don't have to know who it came from. [38]

I care who has [my data]. Like my mom and dad and doctor have my information. That's all the people I want to have [information] I don't want anyone else and some random person. [38]

I feel like you should stop at your healthcare provider or someone that you have a relationship with and no one else needs to know anything about it. If they request information to improve their product and you consent to that, sure, if that's your choice, but not just suck data from everyone that uses the device. [38]

I feel like when it comes to insurance, that they should not be able to just freely have that information. I think that it's something that I would need to be able to give them the information. [38]

If they're [insurance company] using the information to help others right, to help the machine learning or to help the artificial intelligence learn right and adapt and change to help others kind of, the rest of you feel about that I would love nothing more than to have my information used for the betterment of treatment. [38]

I'm generally okay with it [sharing information]as long as I know, and I'm able to in some way like, give consent. I mean, if it's something that they're just taking the numbers, and it has nothing like, there’s no connection with me. It's like, okay, and they're just numbers ultimately like they're not going to have any silly connection to me. But if it's like, they're taking a little bit more about like my profile, whether it be, you know, this is a female. She's nineteen years old, or whatever it may not have my name. But if they're taking a little bit more detailed information, I like to be informed about it, and be able to give consent, if possible. [38]

For one thing, they [the manufacture] need to ask your permission, so you agree or disagree with that, but I feel like they are using it better. All of us that have type one diabetes, because they can, with some of the stuff that happens with us, they can get more knowledge to make better devices. …so use if they ask permission, then you are comfortable with them, using your information. [38]

I care on two points [what information the device collects from me that it is], de-identified and is it [information] sold. [38]

I don't care if it's [my information] used for research purposes, academic research purposes, whatever. But if it is sold to a third party, I want to know that that may happen, and then I want it de-identified. [38]

As long as there's nothing identifiable back to me specifically, then [sharing data is okay], because I think it's actually a good thing if people will provide that data so they can improve things because they can see what works, what doesn't work, [38]

I first want to know with who it's [the patient’s data is] being shared. Is it just, you know, is it just the company? Because again, you're using their portal. Is it being shared with third parties? Is it being shared with the insurance companies? Are they looking at their compliance? So, I think those would be important things to know. Has it been shared with payers? [38]

That was really frustrating. I wanted somebody to ask me, and I never got asked. I didn't tell anybody. So sometimes I want to share that information so that it'll help others and help the improved pumps in the future or whatever the technology is. [38]

Ambiguity regarding responsibility for protecting patient data and obtaining proper consent for AI applications may lead to ethical concerns. [35]

I think at the end of the day, the whole objective is to get family to help us to look after each other and for us to give them the right information. I think we must be very transparent in what we ate… At least you have somebody helping you and not depending on yourself alone. [33]

If you made it such a way that it gives a summary at certain hour checkpoint for instance, by noon, how much have I eaten in terms of calories, what's my goal, how much have I exercised…I’ll be more comfortable rather than a detailed breakdown of everything I do. [33]

It might be suggesting doing something and you’re saying, ‘No because I’m on holiday and I’m going to eat something completely different’. Or, ‘No, I’m in a different time zone so things are changing’. There might be more reasons. Or, ‘No, because I’m ill, so I’m actually going to do this differently instead. My background insulin is going up because I’m ill and I need more’. You might use the ‘no’ reasons more than that, I guess. I guess that could then build a profile based on whether you’re ill, or on holiday. [44]

I mean everyone would look at their personal information differently. I think when you are developing an app, it should cater for all audiences with consensus, and they should be able to revoke it if they feel otherwise. [33]

I think it is quite interesting that the system is able to provide different percentages of achieving optimum blood pressure when different combinations of new drugs are used. However, my question is if plan A gives a score of 48 while plan B gives a score of 45, are these recommendations still clinically relevant? I mean, of course, the situation is more direct in cases with scores such as 98 and 88, then it will make more sense to pick the plan with 98% of likelihood. [40]

**Subtheme 3.2: The Irreplaceable Human Element and Professional Autonomy**

Using AI in patient interactions may diminish the compassionate and personal nature of healthcare, potentially impacting patient satisfaction and confidence. [35]

I mean the kind of personal touch in MI must be done face-to-face. And even in counselling, I believe sometimes tapping on the shoulder, saying something softly, could change the mood as well. [37]

I would like the health coach to be available on the app. The app may be more for daily tracking, right? Then if the health coach, face-to-face, maybe once a month, can talk to me about what my progress is, to give more professional advice, I think that will help me. [37]

I’m not sure if it will really help change patient behaviour. I feel personal touch is still important. [39]

I support AI mobile apps, but I’m skeptical if they can achieve the human touch. We build connections with patients by asking about their lives. This rapport helps change behaviours in diabetes management, which AI might struggle with. [39]

However, there are concerns about privacy, data security, and accessibility for all patients. Some still prefer face-to-face interactions for the emotional support provided by human caregivers. [46]

I would say that I'm as good or even better than the system. I don't feel the need to rely on it; I'll just do what I do. We are all trained endocrinologists, so we trust our judgment because that has been our bread and butter for many years. At the end of the day, we bear the responsibility for our patients, so you know, if the algorithm makes a sound decision, but something unfortunate ever happens to the patient, then it's still our own accountability on the line. [40]

No matter what we still need to see doctor you know. In person, human, we need that human touch to feel better. [41]

after a period of using the data and visualization alone, it is possible that the clinician will not use the chatbot so frequently. [42]

Some of the recommendations go against your clinical judgement. For example, I have two patients and the AI recommendation was to add a beta blocker to someone who doesn't have ischemic heart disease as a second line agent. That's just not something that we would normally do. So have to exercise caution too! [40]

I think that it is important to not make it look like...the fact that [patients are still uncontrolled]...is [because] you [are] a bad physician...I’m tired of that. [34]

Healthcare workers may overly rely on AI-driven analysis, potentially reducing critical thinking and increasing the risk of diagnostic errors. [35]

Healthcare professionals may be hesitant to adopt AI due to concerns about workflow disruptions or job losses. [35]

We must consider language barriers. Someone who doesn’t understand much English might have problems if the app doesn’t include their mother tongue. [39]

**Subtheme 3.3: Increased systemic and caregiver burden**

In terms of time spent, this [module] is secondary especially [since] this is my own problem, so I need to focus on resolving it myself. If I can’t motivate myself, then having someone to motivate me is not going to benefit much.[33]

This app depends on the person…. for my husband [with diabetes], I don't think he will track. He won’t have the diligence to log meals…. I think after a while people may not have that kind of determination to really do it every day consistently. So how do we [family] make them do it? I think that's another thing.[33]

Actually, I’m very independent, doing things on my own. I don't really listen to any counsellor. I know the direction that I wanted to head to...So, I got to do it on my own. I prefer to do it on my own. [37]

If a counsellor wants to motivate me, if I got the time [to listen] and if it’s what I want, I will do. Though I am very open, my time is really not enough so I don’t think I will participate [in-person]. [37]

…He [Father] might find it troublesome. I am not sure if he is willing to [log] every time he takes his medications. [33]

I think in general, yes it should help [with my diabetes] but whether we can find time, that's a different thing. Because right now, between my wife and I, we have our own things we need to settle such as our parents.[33]

If family is going to be involved, it's a burden and if things don’t move the way they are supposed to move, then I’ll feel like I’m not doing my job as a caregiver. My father had cancer, and I was also a caregiver; I know the burden that a caregiver carries and the stress.[33]

Say...I have...10 patients in the morning, and all of them have this alert, and so for all of them, I’m taking...these extra steps to identify barriers...that’s going to take more of my time. [34]

I wouldn’t want a list of 500 patients, because there’s no way that anybody’s going to keep track of that...that would be very difficult. [34]

I think that increasing the cost of care is definitely going to happen...in many systems because of how healthcare is paid for. So if I make a referral...for the patient, and the patient has to go and pay for the social worker [and] dietitian, I’ve just increased the cost of care. [34]

I’m not so into this because different apps are always giving me problems. I have to find the code and speak to people [to learn how to use it]. It's quite frustrating for some of us older folks who are not IT savvy. [37]

For urban patients, access should be no problem. But for rural areas with more districts, it may not be applicable for some patients. [39]

**Theme 4: Technical and educational requirements for successful implementation**

**Subtheme 4.1: Transparency and regulatory compliance**

...if I’m going to use a tool, I want to be able to...click a link [that] will take me to the website and I can just learn more [about] where this is being trained. [34]

Healthcare facilities that implement AI systems must adhere to complex regulatory frameworks to ensure compliance with patient rights and data protection requirements. [35]

There is a risk of a Black Box phenomenon occurring whereby AI algorithms lack transparency and, as such, there is a lack of clarity surrounding the decision-making process. [35]

The lack of transparency may raise ethical questions and create legal challenges when justifying decisions made using AI. [35]

It would be helpful to know why that patient is at risk. And that will make you believe it or not. [34]

I think some sort of report that shows me which factors contributed the most to these alerts may help me even more. [34]

...if the evidence says social work drops the risk by 50% [and] dietitian...drops the risk by 40%, on average, but in my patient, the alert fired because of nutritional concerns, I might choose the dietitian as a first choice because it might have a greater impact for this patient in particular. [34]

...what would be really helpful...would be some sense of the potential impact of each of these, because I’m not going to be able to get my patient to do all six potentially. But if they were organized in such a way to say this step will reduce the risk by this much. That step will reduce the risk by less...then I might be able to prioritize. [34]

I would definitely be open to trialing it but would do it in a quality improvement sort of a mindset where we saw how things were going beforehand and how things were going afterwards. And if it didn’t help me, then I wouldn’t continue using it. [34]

Thorough validation and testing are crucial, as AI algorithms may not consistently perform in different healthcare environments or with diverse equipment. [35]

**Subtheme 4.2: Usability and technical preferences**

The factors would be how useful the tool is, first of all, how validated the tool is and if you can show that...it changes outcomes. [34]

[Adoption] would depend very, very, very heavily on the provider perception of usefulness and usability. [34]

App should be straightforward, not too many buttons or navigations for updates. [39]

It should be very easy to use – user friendly. [39]

Navigating should be easy, with just one click to update. [39]

Interactive elements like simple games can attract attention. Some apps just feed information, but a TikTok-style video could catch patients’ interest. [39]

The AI should adapt itself to the patient’s needs. [39]

Instructions must be clear and understandable by lay persons, regardless of education level. It must be usable by everybody. [39]

It was very helpful [to ask about feature ranges] because I didn’t exactly understand what the difference was between the AI-observed range and the scientific range. Now I understand the key differences between both. [42]

[The conversation with the AI] was quicker than when I Google something and gives me the facts without me needing to read through pages and pages of information to find what I want. [42]

It gives me some key metrics to perform an initial risk status. Clear. I don’t need to scrawl through the patient record to find what I need. [42]

...one of the hard parts about managing diabetes is knowing...they need another agent, and then maybe which agent the insurance might cover...it would be even more beneficial if [the tool told] me these might be suggestive agents to add...for [better] control. [34]

[My use of the tool] would depend a great deal on what resources are actually available to me. [34]

...depending on...what your clinics resources are, if you’re getting alerts for people that you have no ability to help, because you don’t have access to a social worker...that doesn’t feel really good. [34]

Integrating AI into existing healthcare workflows and systems may present technical difficulties that require infrastructure and training investments. [35]

Patients need literacy in technology. They should be able to read, understand instructions, and interpret graphs generated by the app. [39]

Basic technology skills are needed to navigate the apps, use them, and insert data. [39]

They need good technical skills for handling mobiles and gadgets. If they can manoeuvre through the app, they’ll be okay using it. [39]

I’d want to know when and what their last hemoglobin A1c was and when their last appointment was. And then I want to know if they have seen a dietitian in the past and how long ago? [34]

...you could apply the same sort of thing to preventive care to any chronic disease to including depression, hypertension, coronary disease. [34]

...how likely is this person going to follow through on their screenings, [like] getting their mammogram? [34]

One significant issue we encounter after implementing the CDSS is the considerable lag in loading clinical notes. It takes a few minutes to retrieve the clinical notes. So, by that time, I'm typically already engaged in a conversation with the patient, and we may even come up with a plan without the notes being available. In some instances, I can't even see the clinical notes at all. [40]

Our MySejahtera app always lag and very slow. I am afraid how if the diabetes app also be slow like that. Then, the treatment or care plan will be delayed. [41]

...staff should have the means to be able to respond to...this...there would be a lot of a lot of value in having multiple eyes on this to make sure that nobody falls through the cracks. [34]

I don’t think this would be terribly helpful for administrators. Sometimes it’s used punitively. And I don’t think that that’s what we want. [34]

Regarding who should receive this information: “I feel like each location might want to designate that.” [34]

I think [who should receive the information] would be very, very practice dependent...I think giving the information to patients can be really valuable. I think how it’s presented and how it’s framed [is important]. [34]

I think just a lack of context for the patient on why these certain things were ordered would be [a] concern for high alert with the patient...[patients] having no clue what it means could create...panic or some distress in the patient. [34]

Maybe alerts that it is giving you this much insulin, because your blood sugar is high, information about why it's doing what it's doing, so you could learn from what it's doing. [38]

I think if the app keeps prompting me, I’m going to be very tempted to turn off the notifications. Is it possible to tailor it if I only want to receive notifications on the medications because the rest are not as vital to me. [33]

Another thing would be making sure that it’s the right time. So again, if I’m in room with the patient, personally, I don’t want to see these pop up, because I’m probably goal-oriented at that moment where I’m trying to put in something specific and this would just slow me down. [34]

I would be more likely to address it...if it was something I was prompted with when I opened the labs specifically...I’m going there to review their hemoglobin...I’m going there to review their lipids...so if I’m going [to the chart] for that, and...I’m prompted with this, then then I’m going to be more likely to address it right at that moment. [34]

It could predict blood glucose level for sports. So that I can see how the values will change during the activity and I can adapt my eating behaviour and activity to it. [R13; male; aged 26 years] [43]

Prediction is not working at all at the moment. They should connect the Apple Health App to the diabetes App and then tell me how much insulin I have to inject. Or when I do sport the App could give me the alarm a bit earlier and tell me that the values will go down quicker. [R17; male; aged 25 years] [43]

Somewhat help, but not as specific as it needs the be. [42]

Specific to their particular needs. [42]

Have to be taken into consideration when making recommendations. [42]

One size fits all approaches do not work. [42]

To me it is really important what safety features are available, and what's the customization with those safety features. Good distinction sort of where we were headed with the next scenario, because, you essentially said that if it's giving you, and giving you just information or it's making a recommendation you sort of get annoyed about some of those safety features. [38]

No, I just do the test [the device] randomly to make sure everything is running right. But if I don't do it for three weeks, and there has been a problem for 3 weeks, then all my readings are messed up. So, is there a way for an alert to come right on the main screen that something's wrong? [38]

Yeah, I think a hotline or somebody you can talk to if you are encountering any difficulties. [38]

I would like to receive safety information in person. [38]

I think it would be nice to have someone in person to just give me a little breakdown of, “Hey, these are really the most important things that you need to be aware of.” [38]

I'd want [training about safety features] up front. That I'd want when I got the new product. [38]

[information required to safely use the device], probably in a booklet because I hate long startups. I hate to go through a new product and its take up to two hours to get started. [38]

If you're going to give me a written piece of paper, I want to make sure that it's something larger than a 0.5 font size. Because some of these things they put in boxes, they shrink it down to... We have to fit it in a box, and so everything goes down like a millimeter in height. I'm sorry, I just can't read a millimeter height. Not like I used to. Okay? I'm not a Marine or somebody that has great eyesight. I'm serious. You got to have something. That's why I prefer the online where I can at least... or the app, where I can go online and get all that stuff and read it. Because there I can change the font size and actually get something out of it rather than having to hire a 10-year-old kid next door to read it for me. [38]

Ability to turn [alerts and warnings] on and off. Because sometimes you're on schedule and sometimes you're not, it just depends on how busy you are. I mean, everybody's life is different and we all have our own different things like me I am so busy. I get caught up in schoolwork, being a mom and then I'm tutoring sometimes. And I was like, "Did I do this?" And then by the time I'm feeling all woozy, I'm like, "Yeah, my sugar is low. So, let me do something about this before I'll be sitting on the couch." [38]

Patients would benefit from carb counting tools, sugar control trend recording, and connecting glucometer readings directly to the app. [39]

Their renal and liver function profile, medication adherence, and diabetes complication screening. [39]

Early detection of complications is important. Like taking a photo of the leg and sending it to us. AI can analyze for early signs of diabetic foot changes. [39]

It’s important for patients to manage their medication. What’s its function? When to take it? What if I miss a dose? [39]

Patients worry about side effects. If the AI can educate them about potential side effects, that would be good. [39]

We need information on the patient’s lifestyle habits. What are they currently practicing? Are they doing physical activity? What’s their pattern? [39]

The ability to visually present individual risk in a quantitative way through graphical or pictorial means and illustrate the potential changes that may occur after adopting the systems' recommendations would improve information delivery. I personally believe that patients are more inclined to accept the recommendations when they see their risk in a pictorial or a graphical format. [40]

So, the tool helps to reinforce my decision-making. The color-coded recommendations provide a clear visual indication, prompting me to address any discrepancies that may arise between the tool's suggestions and my own clinical plan. In this case, I delve into additional clinical histories that the tool does not have access to and elucidate the rationale behind my decisions. This process enhances my confidence and guides better decision-making during the clinical visit, which can improve the quality of patient care. [40]

Instead of showing six [actionable steps]...you...could [show] fewer options and color [code them]...from most benefit to least benefit. [34]

...there’s a lot of customization that would have to occur on the front end, to make sure that these...action items are clickable [and that] applicable resources [are] available. [34]

This really depends on the operator. For me...if it comes too early, I’ll lose it...So...I feel like [the timing] should be adjustable. That would be best because every provider is very different. [34]

The goal setting will help me achieve what I want to achieve, by giving me better vision and future target, so once I have achieved that target, I can move on to the next target. As I move on, I achieve certain milestones, then from there, it sort of motivates me to continue. [37]

Personally, the best solution for me is, daily when I open the app, it can tell me what I need to do instead of writing so many journals in this app. Better ask me what I want to change and tell me what I can do to improve. I just need a very straightforward instruction. [37]

I would like to see more live ones. I don’t like to read a lot of words or look at cartoons. Sometimes, those things are really misleading, and you don’t understand what they are talking about, like some exercises I saw in graphic forms. [37]

Reminders will help pay attention to your diabetes management, because you might forget and go back to old ways of eating sweet things. But if someone tells me that you must cut your sugar intake, then maybe it will remind me that I shouldn’t be taking so much sugar. It’s like having someone to remind you of…a motivating force. [37]

I like the motivational prompts to be like a two-way communication. So instead of simply telling me ‘Today, you have zero hours of walking’, the reminder can say ‘have you done this already today? Why was it not done yet? Why are you so busy?’ A gentle reminder. Just like talking to your friend who understands me. [37]

…Rewarding will encourage people to change behaviors. If you can exchange points for a voucher, that’s a very good idea, and in addition to step counts, if there are other tasks to increase your points, such as healthy eating, that will motivate people. [37]

I understand the MI through app cannot replace a human, but I’m hoping that it will be better than a chatbot and as human as possible… Just like when you go to a doctor, they give their direct opinions. Certain predefined answers on chatbots at times are not relevant to my concerns. [37]

Will the AI be able to do some size calculation? The Vietnamese pho will be in a huge bowl where the Chinese always serve in smaller bowls. But it can be silly because small bowl can also be bigger and more filled. [33]

AI can be more predictive. If I had nasi lemak for breakfast and salad for lunch, maybe the AI can suggest, based on 2000 calories intake, you would like to eat noodles or rice? [33]

Integration with wearable devices is important. For tracking patient activity, wearable devices are very helpful. [39]

Integration with wearable devices and patient-generated data will help us create more effective, personalized care plans for patients. [46]

I believe wearable devices can help me track my sugar levels more easily. [45]

I want to monitor trends over time easily. [45]

If it can sync with my phone and remind me, it would simplify my routine. [45]

I prefer fewer manual entries in apps. [45]

I want the device to provide actionable feedback, not just numbers. [45]

A summary of daily activity would help me plan better. [45]

A device that integrates with my healthcare provider’s system would be valuable. [45]

Sharing data with my doctor easily is crucial. [45]

I would want to know how to shut it down. [38]

Yeah. I mean, this is going to run off of a base of history that you put in. And so, if you do put in a crazy number, it should say that's not possible or that's too many numbers. I feel like it should. After 90 days of putting information or 30 days of putting information in it should be able to realize that your top number of being high is going to be 300 and your lowest number for the type of person you are, might be 70. And if you put in a crazy number, like 500 and you meant to put 300, it could just say that doesn't make or something like that. [38]

I know there's availability, but I haven't done it, is like a live chat on certain devices, they go to the website and there's a live chat with somebody or video chat with somebody. [38]

I like when the manufacturer has like a 24-hour seven day a week hotline that someone answers the phone and walks them through it. They're really like the experts. [38]

I like that idea like a wallet card almost like maybe having a little wallet card [with all safety alerts and warnings]. [38]

I want to know, is there any action that's required on my part? [38]

I am always on the go so trying to plan ahead is really important to me. It is helpful to know that this might happen in this much time ...I like having predictions. [38]

I just want to know how does it know what to give to me, if [my blood sugars] are going up or down, ...and connections between [devices]. [38]

I feel like there should be an option that says contact your doctor or whatever, if it gets to a point where it’s just, the person just feels a little bit off, they're not agreeing with what's being advised and everything. There should be an option for them or some pop-up or something that says contact your local physician or something like that. Just so that it gives them an out because sometimes people will rely on too much of the information or the app that they forget that, "Oh yeah, I can pick up the phone and call." [38]

**Subtheme 4.3: Segmented education and multichannel support**

I will say that for the design, you might want to make it simple for beginners. You can ask people 10 questions but for others who are not tech-savvy, you can just ask three questions. If someone has a lot of things to tell you, you can ask like 20 questions. [37]

I would prefer seeing, you know, some charts to indicate where I am, so after a certain period, I will know whether I am on the right track. So, a graph or whatever chart will help me. I like more direct outcomes and I want to see them soon. [37]

I think I might want to take part in those caregiver courses because either for myself or if I support my family member, it's going to be useful in the long run so I definitely would want to know if somebody is taking care of me or so I can also help to ease that person's burden. [33]

I believe for families with the elderly with diabetes problems, getting them to take medication regularly and things like that is not easy, it's tough for them. So, they need someone to give them support. so having these courses and resources [will help them]. [33]

A minute or two, not more than that. To get the information across, if there are additional details, then they can look more into it. But the attention spans to capture the person with an attractive heading and for them to click on the video, it should be a minute or two. [33]

It's good to have a guideline for the family member on the things to prepare. For example, if someone doesn’t know how to do foot care, it's good to learn how to manage, otherwise she becomes very stressful. [33]

They need to find validated resources, not just follow any advice. They should know how to find expertise for validated advice, not just marketing. [39]

Patients need to communicate or identify their problems to input into the app. This allows the app to personalize treatment. [39]

They need language skills to read, listen, and understand the information given. [39]

In Malaysia, there’s a diabetes lifestyle program with nice educational content. Patients have free consultations after completing certain modules. [39]

If you're going to use this device like you're really going to need to know this [safety and basic device use information]. So, then you understand what's happening, and how to correctly use it. [38]

8/10, quite a bit more training [then device that provides recommendation]. [38]

I think it would probably better to have some kind of pamphlet that's out, because a pamphlet, usually, it's got a picture or something, or the words are bigger, because the little ones that they insert in your device labels are so small. [38]

I like the interactive as well you know, but I don't know that I would want to go somewhere to do the interaction. I think I'd be happy being on you know, a 15 minute one on one with a case manager or whatever product control specialist whatever to help me set it up, make sure it's running. Make sure I'm comfortable with it and then maybe follow up with me and see if I'm doing all right with it. [38]

I feel like give me all the information, but until I start using it and playing with it, then go back and have some questions, and maybe call the rep that showed me how to use it. Or my doctor, or just getting online and doing the frequently asked questions or blogs. [38]

I personally like the [device] training with the person first, so I can have an opportunity ask questions, and then however else you want to give it to me for support. [38]

The little word paper clip, like make recommendations on how to write your paper, [could be used to give you information about] your pump. [38]

I feel like there needs to be a database for the past experiences of people that have used the app and how it worked for them. I think if there's a database where people with diabetes can relate so as to relate their experiences with the app, so that moving forward, we'll be able to navigate it properly. [38]

Also takes education. So educating providers about what this alert is and what this means and what we what we do with it. [34] [Social worker, FQHC]

I think some people like to know every detail and some don't, so it is really figuring out your level of interest [information needs]. [38]

Individual importance seems quite variable, some trust that since it’s on the market, it must be trustworthy, others seem to treat it like a PubMed article or something, wanting to know literally everything about it. [38]

I think it would probably better to have some kind of pamphlet that's out, because a pamphlet, usually it's got a picture or something, or the words are bigger, because the little ones that they insert in your device labels are so small, you're just like, "Well, I ain't got time to read that. Ain't got time to find my glasses and read that." You don't pay attention to it. But if you got a pamphlet, that's got a nice picture on it, it's got some bold color too, you [will] … read it. [38]

Yeah, I think that [information about how the application calculates dose] should be included in the history, when you're putting together a video, all that information, the calculations, and so on. [38]

Oh my gosh. I would say probably a tutorial kind of thing, especially for the younger generation, since they’re technology savvy. For the older generation, I think that you would have to have the nurses to show them how to do this because some of them don't even understand technology or how to use a phone. [38]

A lot of my experience is just based on my own devices that I wear, but anytime I have a problem with my device or something I can call. I also know I can email them [ the manufacturer]. And I also know that there’s a website where I can go through various problems. [38]

[A place] where we could come in and we could attend and we could ask questions and see demonstrations. [38]

Nice if it [information on maintenance and troubleshooting] was actually a link on the phone on the APP of like “I'm having a problem press this button” and it calls directly the manufacturer of that product or it takes them to the websites. They do a video or even walking through like a decision tree of like what's going on. [38]

Okay well here's the video to help you solve this problem. [38]

I mean, if they're [the patients] up to accessing a website, a website with frequently asked questions, or a place to, you know, type in questions that they have. [38]

[Timing of information provision based on device access] I feel like give me all the information, but until I start using it and playing with it [I won't fully understand], then [come] back when I have some questions, and maybe call the rep that showed me how to use it. [38]

Segmented or tier training I didn't know how to use [my pump] for a while, because we didn't have time to cover everything in that first session, [I would prefer] something I can reference specifically piece by piece rather than this big long “here's everything [training].” Let's sit and go through all of it at once...I lose interest. [38]

I like probably a basic starter guide, of “here's how to do it for a couple of days” and then something that's not too overwhelming, but then maybe something I could click through online on the website, videos or something interactive that goes more in depth on some of the advanced features. [38]

Basic starter, guide of “Here's how to do it” [use your device] for the first couple of days, and then something that's not too overwhelming. But then maybe something I could click through online on the website that would be videos or something interactive of how to go in depth on some of the more advanced features. [38]

Depends on where it is in the cycle. In the beginning, I like a lot of information to be able to hold my hand and walk through it. And then at that point, I just want to touch it and play with it and don't want to be like, “oh, hey, have you tried this?” I'll find that in a second. I'm just absorbing my information as it comes along. [38]

I just want to know at the beginning, explain it to me one time. I don't need to keep learning about it. But from the educator, whoever's giving me the device, I’d rather just talk about it, learn about it, move on. I don't ever read booklets or that kind of thing. I can kind of get lost in it. So if I didn't have a real life person, then just a little video that would maybe explain things [I need to know to safely use the device], like an optional video. [38]

If the recommendations uh are not making sense to you, understand that you know you should contact your health, care provider and get more guidance. So yeah, just so you know, people get lost. Don't forget that they should ask for some outside help and not rely just on that app or that piece of technology. [38]

I do much better with an in person [training], show me how to use device. [38]

Consensus for training media to be digital, also wants available for download for when wifi sucks or something else [is going wrong]. [38]

I feel like give me all the information, but until I start using it and playing with it, then go back and have some questions, and maybe call the rep that showed me how to use it. or my doctor, or just getting online and doing the frequently asked questions or blogs. [38]

I think having both options available because some people will look into it [how the app was created] and take the time to read it. Some people want to actually have the ability to download it and print it and actually read about it. I think having a variety of options available because every person learns differently too. [38]

It's nice to have a video, but it's nice to have since something tangible to review. [38]

[preference for training having] the device to walk through things. Having like the words on the device and say, “you need to do this.” [38]

Before I could even like, put it in when they, when I signed up through the website, and like, made a profile and all that kind of thing, they had like school. There's like orientation modules that you have to complete, where you can like continue on to the other things. [38]

A training [about alerts] for sure, would be beneficial, and then, honestly, like a little cheat card [with all the alerts and their meaning], would be kind of nice if it was just like a little index card or something like that. [38]

I have an app that has a section that you can go to that's like, what's new, and you can go in there and you can navigate and see things. Like that diabetes is drinking from a fire hose, a ton of information all at once. And when you have new products, it's a lot of that. So having a little bit over time or your hands on experience. And then after a while, being able to go back and relearn. That's super helpful, because you'll remember more of it then. [38]

Over the Internet is wonderful, but having it in person, where you say, “now, what about step one way or step two?” [38]

I personally like the [device] training with the person first, so I can have an opportunity ask questions, and then however else you want to give it to me for support. So, I would like, if someone was going to tell me about this app, I would like them to be fully knowledgeable about it, give me the training and then give me something in writing to follow-up that basically says the same thing or more than they just said, and then make themselves available to me to ask questions. [38]

There's too much information for me [with all the testing studies]. I don't really [need to know]... Try to keep things simple. [38]

So having it accessible is really nice because then you can do a lot of it on your own. [38]

If they're [the patients] up to accessing a website, a website with frequently asked questions, or a place to, you know, type in questions that they have, I think most folks would have some kind of access to that, but we take care of pretty poor folks, so I know how much it costs to keep my own Internet going. [38]

I don't remember [troubleshooting information I received during initial training]. For the most part [I] feel like just having something I can look up myself, not having to go through calling 7 people and being on the phone [waiting]. Sometimes getting the right person is really frustrating. [38]

If you have a 24-hour service, that's going to require some sort of a payment I would think. And with an app like that, especially for the older group who is Medicaid or whatever, really trying to manage costs and everything. So, would that have to come into play? I mean, would that deter people from doing something like this? [38]

What would be kind of cool if it was possible, is, do like a zoom thing, if necessary, where you could, you know, meet with them and be able to like literally hold up your pump, or whatever. If it's having a problem, you'd be like, “This is what's happening. This is the alert that's coming. What does this mean?” [38]

Maybe it is in some paperwork and I don't read the whole pamphlet every time, but I find it more helpful to have something pop up in the app itself, so that I have to look at it rather than just putting it in like some big long list of, “Here's some possibilities of what might happen.” [38]

I personally like the [device] training with the person first, so I can have an opportunity ask questions, and then however else you want to give it to me for support. So, I would like, if someone was going to tell me about this app, I would like them to be fully knowledgeable about it, give me the training and then give me something in writing to follow-up that basically says the same thing or more that they just said, and then make themselves available to me to ask questions. [38]

Maintenance and troubleshooting information should include] professional training, and we need a brochure. [38]

That would be super helpful to you [the patient] if [information was] already there in the APP that could be built in. [38]

I think the manufacturer should have a 24-hour seven day a week troubleshooting number. [38]

So, you would like the step by step within the device, but you want the ability to turn it off once you have figured it out. [38]

We also need to have some videos that you know are Youtube videos [pop-up information videos] that also can hide [be in the background for when needed], there if you need it or you're confused. [38]

Basically, the same [in-person during the training and video alerts as back-up]. [38]

I'd want both a chat bot in the app, and maybe contact information, for example, a phone number or an email address to act as a backup [for information communication]. [38]

It'd be nice if I had a separate app that could say, type in that error code and say, what do I do? If I can't find an online reference as to what I need to do or some kind of video as to how to fix it, then I'd better have an 800 number, or I can actually talk to a human being and say, "We got a problem, Houston." [38]

If I yeah again, within the algorithm, I would hope that if something's not going right, or the patient's perhaps doing something wrong, or they can't figure it out that they would have like a frequently asked questions. [38]
